# Development of therapeutic antibodies for the treatment of diseases

**DOI:** 10.1186/s43556-022-00100-4

**Published:** 2022-11-22

**Authors:** Zeng Wang, Guoqing Wang, Huaqing Lu, Hongjian Li, Mei Tang, Aiping Tong

**Affiliations:** 1 State Key Laboratory of Biotherapy and Cancer Center, Research Unit of Gene and Immunotherapy, Chinese Academy of Medical Sciences, Collaborative Innovation Center of Biotherapy, West China Hospital, Sichuan University, Chengdu, China; 2 Department of Neurosurgery, West China Medical School, West China Hospital, Sichuan University, Chengdu, China; 3 Institute for Immunology and School of Medicine, Tsinghua University, Beijing, China

**Keywords:** Immunotherapy, Antibody drugs, Phage display libraries, Transgenic mice, Single B-cell, AI-assisted antibody discovery

## Abstract

Since the first monoclonal antibody drug, muromonab-CD3, was approved for marketing in 1986, 165 antibody drugs have been approved or are under regulatory review worldwide. With the approval of new drugs for treating a wide range of diseases, including cancer and autoimmune and metabolic disorders, the therapeutic antibody drug market has experienced explosive growth. Monoclonal antibodies have been sought after by many biopharmaceutical companies and scientific research institutes due to their high specificity, strong targeting abilities, low toxicity, side effects, and high development success rate. The related industries and markets are growing rapidly, and therapeutic antibodies are one of the most important research and development areas in the field of biology and medicine. In recent years, great progress has been made in the key technologies and theoretical innovations provided by therapeutic antibodies, including antibody–drug conjugates, antibody-conjugated nuclides, bispecific antibodies, nanobodies, and other antibody analogs. Additionally, therapeutic antibodies can be combined with technologies used in other fields to create new cross-fields, such as chimeric antigen receptor T cells (CAR-T), CAR-natural killer cells (CAR-NK), and other cell therapy. This review summarizes the latest approved or in regulatory review therapeutic antibodies that have been approved or that are under regulatory review worldwide, as well as clinical research on these approaches and their development, and outlines antibody discovery strategies that have emerged during the development of therapeutic antibodies, such as hybridoma technology, phage display, preparation of fully human antibody from transgenic mice, single B-cell antibody technology, and artificial intelligence-assisted antibody discovery.

**Supplementary Information:**

The online version contains supplementary material available at 10.1186/s43556-022-00100-4.

## Introduction

An antibody, also known as an immunoglobulin (Ig), is a glycoprotein produced by differentiated B lymphocytes, which makes up the humoral component of the adaptive immune system in response to exposure to pathogens. They comprise two larger heavy (H) chains (Igh) and two light (L) chains (Igκ or Igλ) linked by covalent disulfide bonds to form a tetrameric structure. Antibodies have been widely studied because of their high specificity and affinity with very high efficiencies in a lot of clinical diagnostic and therapeutic applications. Antibody-based protein derivatives, including recombinant monoclonal antibody (mAb), antibody–drug conjugate (ADC), bispecific antibody (BsAb), antibody fragments, and Fc-fusion protein, establish an important part of therapeutic agents.

The first approved-for-use therapeutic antibody (Muromonab-CD3) in 1986 was a mouse hybridoma mAb. This antibody, to some extent, was a straightforward result of the hybridoma technique advanced by Kohler and Milstein in 1975 [1]. Since hybridoma technology was discovered, antibody-based therapies have continuously derived [2]. Murine mAbs in patient bodies as if they were a foreign substance will cause human anti-mouse antibodies (HAMAs) because of no human species component and strong immunogenicity [3]. The muromonab-CD3 elicited a HAMA response that caused neurotoxicity in patients who received it [4]. To overcome these problems, techniques were matured to convert murine antibodies into structures similar to human antibodies while retaining their binding properties. Abciximab, known as an anti-GPIIb/IIIa Fab, was first approved for use chimeric antibody in 1994 by the United States (US) Federal Drug Administration (FDA) for the treatment of platelet aggregation [5]. Although the chimeric antibody exhibited a resolved HAMA response to a certain extent, its variable region is still of murine origin, there is the possibility of inducing HAMA, and further optimization is needed. Another new progress was the complementary-determining region (CDR) grafting technique resulted in humanized antibodies [6]. Daclizumab, an anti-IL-2 receptor antibody, was the first humanized mAb approved for use by the US FDA in 1997 for the prevention of transplant rejection [7, 8]. There is a well-known therapeutic antibody, such as bevacizumab, which targets vascular endothelial growth factor (VEGF), has been approved for use as a first-line treatment for metastatic colorectal cancer in 2004 [9]. In order to continue to reduce the immunogenicity of humanized antibodies, an important discovery technology that has been used to generate completely human mAbs was developed by Sir Gregory P. Winter in 1990 [10]. It was based on the phage display technique, inserting different exogenous genes into the phage vector. With the proliferation of phage, the foreign protein will be displayed on the surface of the phage to form a phage library [11]. Adalimumab (Humira), an antitumor necrosis factor α (TNFα) fully human antibody [12], was approved for use in the treatment of cancer [13] and autoimmunity [14–16] in 2002 by the US FDA. Preparation of fully human antibody from transgenic mice represents another technology [17] that is used for generating fully human mAbs. Panitumumab, an anti-epidermal growth factor receptor (EGFR) fully human antibody [18, 19], and nivolumab, targets programmed cell death protein 1 (PD-1), a fully human IgG4 mAb [20, 21], which were both produced through a transgenic humanized mouse antibody platform.

**Fig. 1:**
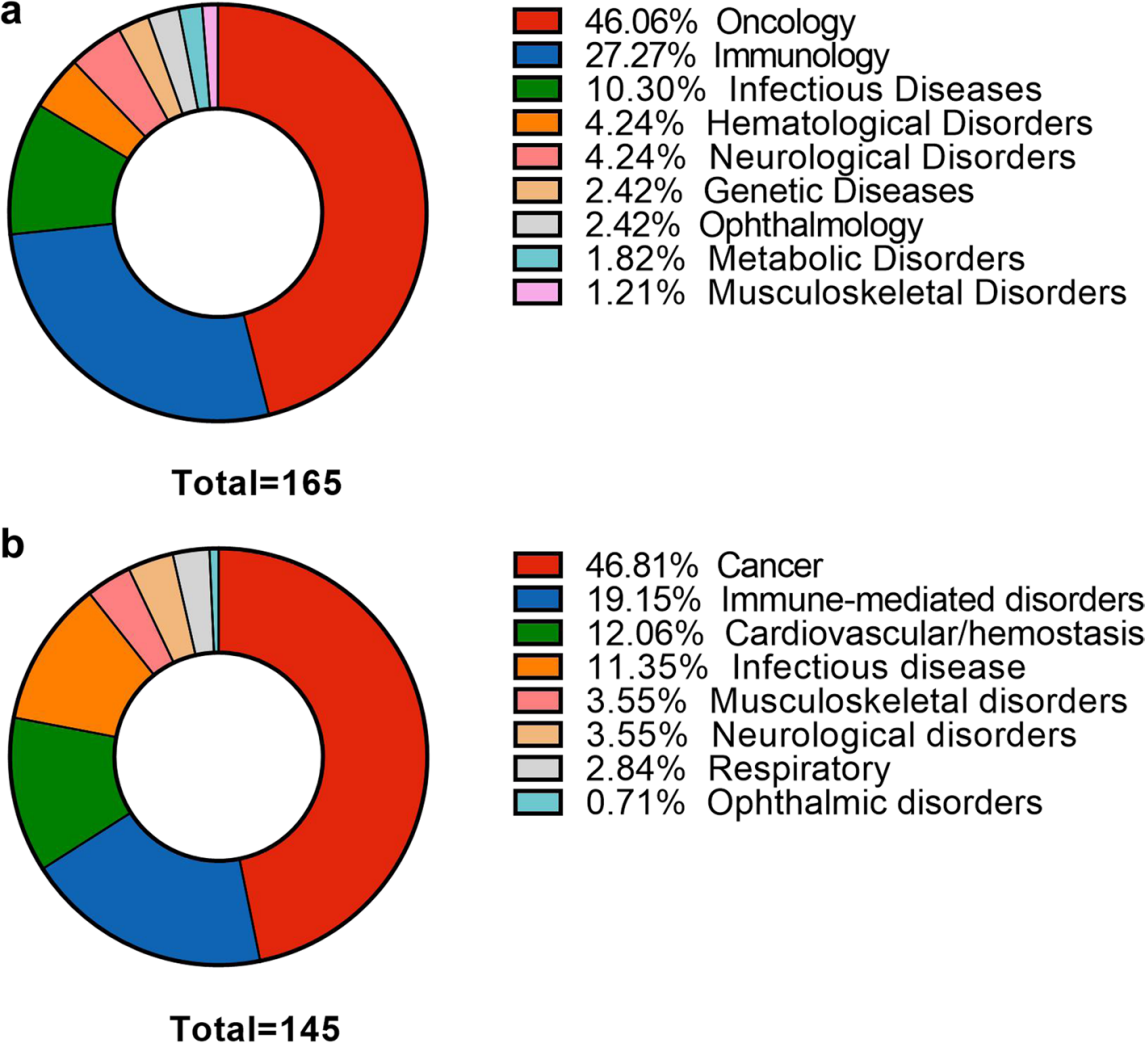
Primary indications for antibody therapeutics are approved across the globe and in late-stage clinical studies sponsored by commercial firms. **a** Primary indications for antibody therapeutics are approved across the globe. Immune-mediated disorders category includes asthma, systemic lupus erythematosus, rheumatoid arthritis, etc.; the genetic disorders are Muckle-Wells syndrome, X-linked hypophosphatemia, hereditary angioedema attacks, and homozygous familial hypercholesterolemia. Figure based on data publicly available (www.antibodysociety.org/antibody-therapeutics-product-data/.) as of July 1, 2022, total = 165. **b** Primary indications for antibody therapeutics in late-stage clinical studies. “Late-stage” is defined as pivotal Phase II, Phase II/III, or Phase III studies. Immune-mediated disorders category includes allergy and asthma; respiratory includes chronic obstructive pulmonary disease. Figure based on data publicly available (www.antibodysociety.org/antibody-therapeutics-product-data/.) as of May 1, 2022, total = 145

**Fig. 2:**
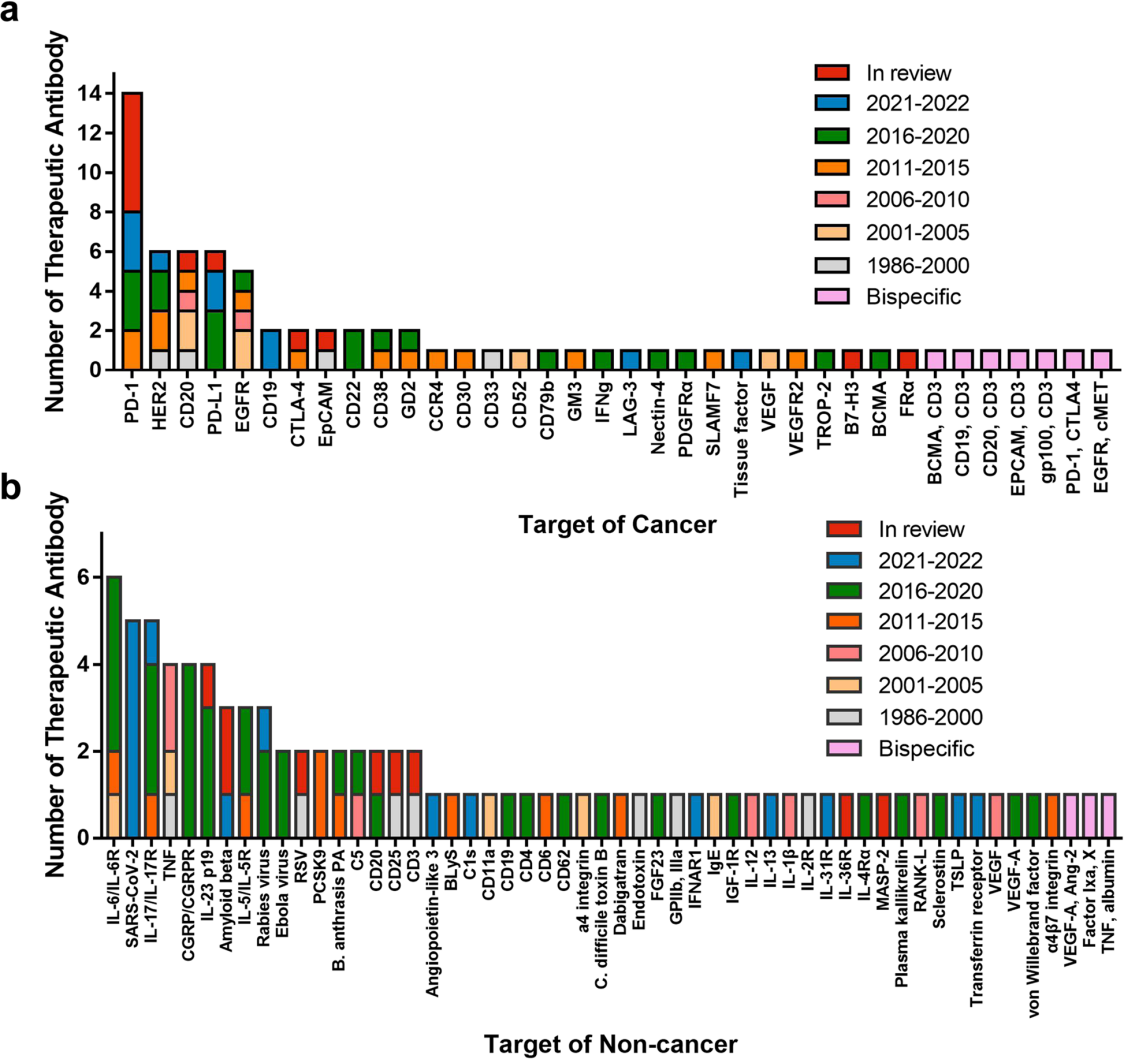
Targets for antibody therapeutics approved or in regulatory review globally for diseases. **a** targets for cancer and **b** targets for non-cancer. TSLP, thymic stromal lymphopoietin. Figure based on data publicly available (www.antibodysociety.org/antibody-therapeutics-product-data/.) as of July 1, 2022, total = 165

With decades of development, therapeutic antibody drugs have increased in number, variety, and categories. By April 2021, with the approval of the dostarlimab antibody targeting PD-1, the number of antibody drugs has reached 100 from 50 in just over 6 years [22]. Antibody drugs now account for approximately one-fifth of new FDA-approved drugs annually. According to data published by the Antibody Society, as of July 1, 2022, there were 165 therapeutic antibody drugs approved or in regulatory reviews across the globe. Antibodies have become an important component of modern biomedicine for the treatment of cancer, immune disease, infectious disease, blood system disease, nervous system disease, genetic disease, and some other diseases. We analyzed the data associated with these products and found that nearly half (46.06%) were treatment for cancer, 27.27% for the immune-mediated disorder, and 10.30% for infectious disease applications; the number used for treating infectious disease increased with the emergence of COVID-19 (Fig. 1a). Of those used to treat cancer (*n* = 75), the most frequent targets include PD-1, human epidermal growth factor receptor 2 (HER-2), CD20, and programmed cell death-ligand 1 (PD-L1). Of those used to treat diseases other than cancer (*n* = 90), the number of therapeutic antibodies targeting SARS-CoV-2 has increased the fastest in the past two years. The other common targets include TNF, interleukin (IL)-6 or IL-6 receptor, IL-17 or IL-17 receptor, and calcitonin gene-related peptide (CGPR) or CGPR receptor (Fig. 2).

In recent years, ADC [23] and BsAb, as new types of antibody drugs, are the hot fields of current pharmaceutical research and development, and their approvals have increased each year [24]. In addition, antibody formats such as Fab, single-chain variable fragment (scFv), and nanobody (VHH) technologies, have gradually started to be approved for marketing or have entered later-stage clinical trials, research, and development.

This review mainly summarizes the development of therapeutic antibodies for the treatment of diseases in approval and clinical trial, including monoclonal antibodies, antibody–drug conjugates, bispecific antibodies, and antibody fragments. We also select the COVID-19 disease as a representative to briefly describe the development and limitations of therapeutic antibodies in the treatment of COVID-19. The preclinical development of therapeutic antibodies can be roughly divided into target discovery and verification, antibody preparation and screening, pilot production and quality control of antibodies, drug efficacy, pharmacokinetics, safety assessment and declaration, etc. We focus on several key technologies for the early preparation and screening of antibody drugs, including hybridoma technology, phage display human antibody libraries, human antibodies from transgenic mice, single B-cell antibody technology, and AI-assisted antibody discovery.

## Monoclonal antibodies (mAbs)

A monoclonal antibody is generated from a single B lymphocyte and has a high degree of specificity binding to a certain epitope of an antigen. The heavy chain and light chain variable domains, VH and VL) are responsible for the specific binding to the antigen to inhibit or neutralize the antigen [25]. There are three hypervariable regions known as CDRs in VH and VL. This constant region also referred to as the Fc region, determines the class of the mAb and its function [26]. The Fc region determines the effector functions of antibodies, including antibody-dependent cell cytotoxicity (ADCC) [27], antibody-dependent cellular phagocytosis (ADCP) [28], and complement-dependent cytotoxicity (CDC) [29]. Therefore, therapeutic monoclonal antibodies were developed as a result to block or inhibit the activity of a target enzyme, a cell surface transporter, or a signaling molecule and have been used in cancer immunotherapy and to treat severe viral infections. Current applications of mAbs include the treatment of other severe, nonmalignant diseases including atopic dermatitis, asthma, hypercholesterolemia, osteoporosis, migraine headaches, and bacterial infections (such as anthrax).

### Monoclonal antibodies currently approved and market

**Fig. 3:**
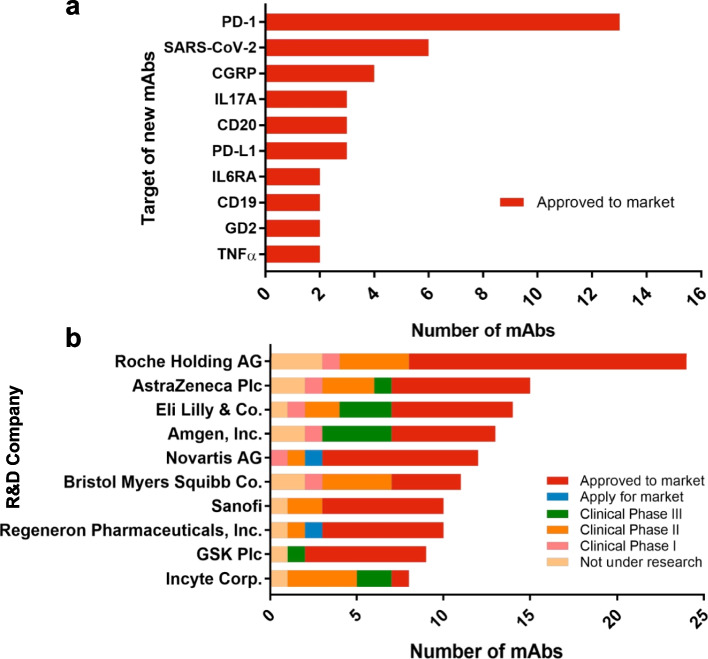
Histogram of the number of mAbs for the top ten targets and R&D companies. **a** The top ten targets of mAbs approved to market for use from 2021. Data were collected from January 1, 2021, to August 1, 2022. **b** The top ten institutions and the distribution of their drug R&D stages. R&D, research and development. Figure based on data publicly available as of August 1, 2022, and available at https://pharmsnap.zhihuiya.com/

By the end of July 2022, a total of 123 mAbs were approved for marketing (Table 1). Globally, 65 new mAbs were approved from 2021 onward. Thirteen antibodies targeting PD-1 have been approved, with PD-1 being the most developed target among those approved new mAbs (Fig. 3a). Notably, pucotenlimab, a humanized anti-PD-1 mAb, was successfully approved for use in China on July 19, 2022. Preclinical data (CTR20180125) suggest that it exerts antitumor effects [30]. Pembrolizumab, a humanized anti-PD-1 antibody approved for use by the US FDA in 2014, was used for treating advanced solid tumors [31, 32], such as non-small cell lung cancer (NSCLC) [33–35], early triple-negative breast cancer (TNBC) [36] and malignant pleural diseases [37]. Nivolumab, an anti-PD-1 mAb that was also approved for use by the US FDA in 2014 as well, was assessed as a treatment for recurrent or metastatic squamous-cell carcinoma of the head and neck [20], advanced renal-cell carcinoma [38], and nonsquamous NSCLC [39]. Additionally, it works on Glioblastoma Multiforme since the PD-L1 protein is abundant on its surface [40]. A lot of randomized clinical trials indicated that NSCLC patients who were treated combination with nivolumab with pembrolizumab showed higher overall survival than those treated with docetaxel [41]. Ustekinumab (Stelara) is a human mAb that binds to the p40 subunit common to both interleukin (IL)-12 and IL-23 [42], which are inflammatory cytokines implicated in Crohn's disease pathophysiology [43]. It was approved as an induction and maintenance therapy for Crohn’s disease [44] and ulcerative colitis [45]. Chimeric antibodies, such as rituximab [46] were the first FDA-approved mAb for use during the treatment of lymphoma patients [47]. Ublituximab [48], which targets CD20, a novel and glycoengineered anti-CD20 mAb with single-agent activity in patients with relapsed chronic lymphocytic leukemia (CLL) [49]. Currently, ublituximab is in review for FDA approval, for the treatment of multiple sclerosis (MS), relapsing–remitting MS [50], lymphoma, and diffuse large B-cell (DLBCL) [51]. Obexelimab is a chimeric mAb that targets the CD19 molecule and simultaneously binds the Fcγ receptor IIb (FcγRIIb), the only inhibitory Fcγ receptor that is expressed on the surface of B cells [52, 53], to treat the autoimmune disease systemic lupus erythematosus (SLE) [54]. It has been used in phase II clinical trials for the treatment of 104 patients with moderate to severe SLE. Naxitamab [55] (DANYELZA®) is a humanized (IgG1) anti-GD2 (hu3F8) mAb that was developed for the treatment of neuroblastoma [56], osteosarcoma [57] and other GD2-positive cancers [58]. It was granted accelerated approval for marketing as treatment drug by the U.S. FDA in 2020 [59]. Isatuximab (Sarclisa®) is an anti-CD38 mAb [60] for use in the treatment of adults with multiple myeloma (MM) [61, 62].

**Table 1:** Monoclonal antibodies first approved or undergoing regulatory review^b^

Therapeutic Area	INN^a^	Drug Code(s)^a^	Target	Format	First global approval
Immunology	Muromonab-CD3	Orthoclone Okt3	CD3	Murine	US, 1986
Immunology	Efalizumab	Raptiva	CD11a	Humanized	US, 2003
Immunology	Daclizumab	Zinbryta; Zenapax	IL-2R	Humanized	US, 1997
Immunology	Basiliximab	Simulect	IL-2R	Chimeric mouse/human	US, 1998
Immunology	Infliximab	Remicade	TNF	Chimeric mouse/human	US, 1998
Immunology	Adalimumab	Humira	TNF	Human	US, 2002
Immunology	Omalizumab	Xolair	IgE	Humanized	US, 2003
Immunology	Natalizumab	Tysabri	a4 integrin	Humanized	US, 2004
Immunology	Ustekinumab	Stelara	IL-12/23	Human	EU, 2009
Immunology	Golimumab	Simponi	TNF	Human	US, 2009
Immunology	Tocilizumab	Actemra	IL-6R	Humanized	Japan, 2005
Immunology	Belimumab	Benlysta	BLyS	Human	US, 2011
Immunology	Siltuximab	Sylvant	IL-6	Chimeric mouse/human	US, 2014
Immunology	Vedolizumab	Entyvio	α4β7 integrin	Humanized	US, 2014
Immunology	Secukinumab	Cosentyx	IL-17a	Human	Japan, 2014
Immunology	Mepolizumab	Nucala	IL-5	Humanized	US, 2015
Immunology	Ixekizumab	Taltz	IL-17a	Humanized	US, 2016
Immunology	Reslizumab	Cinqaero	IL-5	Humanized	US, 2016
Immunology	Brodalumab	Kyntheum	IL-17R	Human	Japan, 2016
Immunology	Dupilumab	Dupixent	IL-4Rα	Human	US, 2017
Immunology	Guselkumab	TREMFYA	IL-23 p19	Human	US, 2017
Immunology	Sarilumab	Kevzara	IL-6R	Human	Canada, 2017
Immunology	Ocrelizumab	OCREVUS	CD20	Humanized	US, 2017
Immunology	Benralizumab	Fasenra	IL-5Rα	Humanized	US, 2017
Immunology	Tildrakizumab	Ilumya	IL-23 p19	Humanized	US, 2018
Immunology	Risankizumab	Skyrizi	IL-23 p19	Humanized	Japan, 2019
Immunology	Inebilizumab	Uplizna	CD19	Humanized	US, 2020
Immunology	Satralizumab	Enspryng	IL-6R	Humanized	Canada, 2020
Immunology	Tralokinumab	Adtralza	IL-13	Human	EU, 2021
Immunology	Teplizumab	(Pending)	CD3	Humanized	In review
Immunology	Anifrolumab	Saphnelo	IFNAR1	Human	US, 2021
Immunology	Inolimomb	(Pending)	CD25	Murine	In review
Immunology	Bimekizumab	Bimzelx	IL-17A	Humanized	EU, 2021
Immunology	Sutimlimab,	Enjaymo	C1s	Humanized	US, 2022
Immunology	Ublituximab	(Pending)	CD20	Chimeric	In review
Immunology	Tezepelumab	Tezepelumab	Thymic stromal lymphopoietin	Human	US, 2021
Immunology	Spesolimab	(Pending)	IL-36R	Humanized	In review
Immunology	Itolizumab	Alzumab	CD6	Humanized	India, 2013
Immunology	Netakimab	Efleira	IL-17	Humanized	Russia, 2019
Immunology	Levilimab	Ilsira	IL-6R	Human	Russia, 2020
Immunology	Olokizumab	ARTLEGIA	IL-6	Humanized	Russia, 2020
Immunology	Nemolizumab	Mitchga	IL-31 receptor alpha	Humanized	Japan, 2022
Immunology	Mirikizumab	(Pending)	IL-23p19	Humanized	In review
Oncology	Edrecolomab	Panorex	EpCAM	Murine	Germany, 1995
Oncology	Rituximab	MabThera, Rituxan	CD20	Chimeric mouse/human	US, 1997
Oncology	Trastuzumab	Herceptin	HER-2	Humanized	US, 1998
Oncology	Cetuximab	Erbitux	EGFR	Chimeric mouse/human	Switzerland, 2003
Oncology	Bevacizumab	Avastin	VEGF	Humanized	US, 2004
Oncology	Panitumumab	Vectibix	EGFR	Human	US, 2006
Oncology	Ofatumumab	Arzerra	CD20	Human	US, 2009
Oncology	Ipilimumab	Yervoy	CTLA-4	Human	US, 2011
Oncology	Pertuzumab	Perjeta	HER-2	Humanized	US, 2012
Oncology	Obinutuzumab	Gazyva, Gazyvaro	CD20	Humanized	US, 2013
Oncology	Racotumomab	Vaxira	GM3	Murine	Cuba, 2013
Oncology	Ramucirumab	Cyramza	VEGFR2	Human	US, 2014
Oncology	Nivolumab	Opdivo	PD1	Human	US, 2014
Oncology	Pembrolizumab	Keytruda	PD1	Humanized	US, 2014
Oncology	Alemtuzumab	Lemtrada	CD52	Humanized	US, 2001
Oncology	Necitumumab	Portrazza	EGFR	Human	US, 2015
Oncology	Dinutuximab	Unituxin	GD2	Chimeric mouse/human	US, 2015
Oncology	Daratumumab	Darzalex	CD38	Human	US, 2015
Oncology	Elotuzumab	Empliciti	SLAMF7	Humanized	US, 2015
Oncology	Olaratumab	Lartruvo	PDGFRα	Human	US, 2016
Oncology	Atezolizumab	Tecentriq	PD-L1	Humanized	US, 2016
Oncology	Avelumab	Bavencio	PD-L1	Human	US, 2017
Oncology	Durvalumab	Durvalumab	PD-L1	Human	US, 2017
Oncology	Mogamulizumab	Poteligeo	CCR4	Humanized	Japan, 2012
Oncology	Cemiplimab	Libtayo	PD-1	Human	US, 2018
Oncology	Emapalumab	Gamifant	IFNg	Human	US, 2018
Oncology	Trastuzumab	Enhertu	HER-2	Humanized	US, 2019
Oncology	Isatuximab	Sarclisa	CD38	Chimeric mouse/human	US, 2020
Oncology	Tafasitamab	Monjuvi	CD19	Humanized	US, 2020
Oncology	Dostarlimab	Jemerli	PD-1	Humanized	EU, 2021
Oncology	Retifanlimab	(Pending)	PD-1	Humanized	In review
Oncology	Toripalimab	Tuoyi	PD-1	Humanized	In review
Oncology	Sintilimab	Tyvyt	PD-1	Human	In review
Oncology	Penpulimab	(Pending)	PD-1	Humanized	In review
Oncology	Tislelizumab	(Pending)	PD-1	Humanized	In review
Oncology	Relatlimab	Opdualag	LAG-3	Human	US, 2022
Oncology	Tremelimumab	(Pending)	CTLA-4	Human	In review
Oncology	Nimotuzumab	TheraCIM	EGFR	Humanized	Cuba, 2002
Oncology	Camrelizumab	AiRuiKa	PD-1	Humanized	China, 2019
Oncology	Inetetamab	Cipterbin	HER-2	Humanized	China, 2020
Oncology	Sugemalimab	Cejemly®	PD-L1	Human	China, 2021
Oncology	Zimberelimab	-	PD-1	Human	China, 2021
Oncology	Prolgolimab	Forteca	PD-1	Humanized	Russia, 2020
Oncology	Geptanolimab	-	PD-1	Humanized	Regulatory review in China
Oncology	Ripertamab	-	CD20	Chimeric	Regulatory review in China
Oncology	Serplulimab	-	PD-1	Humanized	China, 2022
Oncology	Socazolimab	-	PD-L1	Human	Regulatory review in China
Infectious Diseases	Nebacumab	Centoxin	Endotoxin	Human	Germany, 1991
Infectious Diseases	Palivizumab	Synagis	RSV	Humanized	US, 1998
Infectious Diseases	Raxibacumab	(Pending)	B. anthrasis PA	Human	US, 2012
Infectious Diseases	Obiltoxaximab	Anthim	B. anthrasis PA	Chimeric mouse/human	US, 2016
Infectious Diseases	Bezlotoxumab	Zinplava	Clostridium difficile enterotoxin B	Human	US, 2016
Infectious Diseases	Ibalizumab	Trogarzo	CD4	Humanized	US, 2018
Infectious diseases	Regdanvimab	Regkirona	SARS-CoV-2	Human	Republic of Korea, 2021
Infectious diseases	Sotrovimab	Xevudy	SARS-CoV-2	Human	Australia, 2021
Infectious diseases	Nirsevimab	(Pending)	RSV	Human	In review
Infectious disease	-	RabiShield	Rabies virus G glycoprotein	Human	India, 2016
Infectious disease	Ormutivimab	-	Rabies virus surface glycoprotein 4	Human	China, 2022
Hematological Disorders	Eculizumab	Soliris	C5	Humanized	US, 2007
Hematological Disorders	Ravulizumab	Ultomiris	C5	Humanized	US, 2018
Hematological Disorders	Crizanlizumab	Adakveo	CD62 (aka P-selectin)	Humanized	US, 2019
Hematological Disorders	Narsoplimab	(Pending)	MASP-2	Human	In review
Neurological disorders	Erenumab	Aimovig	CGRP receptor	Human	US, 2018
Neurological disorders	Galcanezumab	Emgality	CGRP	Humanized	US, 2018
Neurological disorders	Fremanezumab	Ajovy	CGRP	Humanized	US, 2018
Neurological disorders	Eptinezumab	VYEPTI	CGRP	Humanized	US, 2020
Neurological disorders	Aducanumab	ADUHELM	Amyloid beta	Human	US, 2021
Neurological disorders	Lecanemab	(Pending)	Amyloid beta protofibrils	Humanized	In review
Neurological disorders	Donanemab	(Pending)	Amyloid beta, N3pG	Humanized	In review
Genetic Diseases	Canakinumab	Ilaris	IL-1β	Human	US, 2009
Genetic Diseases	Burosumab	Crysvita	FGF23	Human	EU, 2018
Genetic Diseases	Lanadelumab	Takhzyro	Plasma kallikrelin	Human	US, 2018
Genetic Diseases	Evinacumab	Evkeeza	Angiopoietin-like 3	Human	US, 2021
Musculoskeletal Disorders	Denosumab	Prolia	RANK-L	Human	EU, 2010
Musculoskeletal Disorders	Romosozumab	Evenity	Sclerostin	Humanized	Japan, 2019
Other	Evolocumab	Repatha	PCSK9	Human	EU, 2015
Other	Alirocumab	Praluent	PCSK9	Human	US, 2015
Other	Teprotumumab	Tepezza	IGF-1R	Human	US, 2020

^a^Some antibodies have not been found INN (International Non-Proprietary Name) or Drug Code

^b^Table data based on publicly available antibody society (www.antibodysociety.org/antibody-therapeutics-product-data/.)

It is expected that the mAb market will grow at a rapid pace in the near future, as there is an adequate pipeline of products already available. At present, antibody drugs are among the most popular products available. Among the top 50 drugs based on drug sales in 2021, 22 are antibody drugs. Among them, the number one antibody drug is adalimumab (Humira) [63], a popular drug developed by AbbVie, with yearly sales exceeding $20 billion. It is second only to the Comirnaty COVID-19 vaccine developed by Pfizer/BioNTech [64–66], with sales reaching an astonishing $59.1 billion that year. The second-ranked antibody drug is pembrolizumab (Keytruda) [67, 68], and a total of $17 billion in annual sales has been reported. The global antibody drug market has maintained a growth rate of over 10% for the past eight consecutive years, surpassing $200 billion for the first time in 2021. As an increasing number of antibody drugs are approved, it is expected that the share of antibody drugs will increase. Despite this high growth potential, new companies are unlikely to lead in research and development (R&D) efforts; seven companies currently lead R&D efforts, including Roche Holding AG (*n* = 16), Novartis AG (*n* = 9), AstraZeneca Plc (*n* = 8), Eli Lilly & Co. (*n* = 7), Sanofi (*n* = 7), Regeneron Pharmaceuticals, Inc.(*n* = 7), GSK Plc (*n* = 7), Amgen, Inc. (*n* = 6), and Bristol Myers Squibb Co. (*n* = 4), with other companies comprising the remainder (Fig. 3b).

### Monoclonal antibodies currently in late-stage clinical trials

According to the Antibody Society, as of May 1, 2022, there were over thousands of antibody drugs in clinical trials worldwide. Of these studies, about 90% of these studies are early-stage assessments of safety and preliminary efficacy in patient populations (Phase I, I/II, or Phase II). Of these, 145 therapeutic antibodies are in late-stage clinical trials, including 98 monoclonal antibodies (Supplementary Table 1). No single therapeutic area dominated the trials for these antibodies, but cancer accounted for 46.81% of them (Fig. 1b). The data collected from completed and ongoing clinical trials were obtained through ClinicalTrials.gov. It was discussed in the last update regarding the clinical evidence supporting the efficacy of monoclonal antibodies.

TIGIT inhibitor tiragolumab may be effective in treating solid tumors when combined with the PD-L1 inhibitor atezolizumab. Multiple solid malignancies-most notably non-small cell lung cancer-had statistically significant responses in phase I and II trials of the agent [69]. A comparison of tigolumab plus atezolizumab against placebo plus atezolizumab in patients with chemotherapy-naive, PD-L1-positive, recurrent or metastatic NSCLC demonstrated a clinically meaningful improvement in objective response rate and progression-free survival [70]. SKYSCRAPER-07 will determine if tiragolumab plus atezolizumab combination therapy provides superior clinical benefit to atezolizumab monotherapy or placebo in patients with unresectable esophageal squamous cell carcinoma (NCT04543617) [71]. The humanized mAb TQB2450 is a novel anti-PD-L1 antibody that has shown promising results in combination with anlotinib for the treatment of multiple cancers. In patients with localized or metastatic soft-tissue sarcomas, TQB2450 was shown to be effective and safe when combined with anlotinib in a Phase II trial [72]. TQB2450 plus anlotinib provides promising anti-cancer activity while causing manageable toxic effects in patients with platinum-resistant and -refractory ovarian cancer [73] and pretreated advanced biliary tract cancer [74]. The findings are being further validated in a phase III randomized controlled trial (NCT04236362). It has been developed as a monoclonal antibody of high affinity against human neonatal Fc receptors (FcRn) to reduce pathogenic IgG in diseases caused by autoimmune and alloimmune responses. It may provide clinical benefit in patients with generalized myasthenia gravis (NCT03971422) and immune thrombocytopenia (NCT02718716) were generally well tolerated [75, 76]. As an effective treatment for adult patients with moderate to severe atopic dermatitis, lebrikizumab, a novel monoclonal antibody targeting IL-13, was demonstrated to be safe and effective with rapid, dose-dependent efficacy [77] (NCT03443024).

## Antibody–drug conjugates (ADCs)

Currently, in addition to mAb, ADC is one of the fastest-growing type segments of antibody drugs. To date, of the 165 antibody creations that have been approved for use or are under review globally, approved ADCs account for 13 (Table 2), and antibody-conjugated radioactive elements and immunotoxins each account for 2. Overall, there are a total of 23 antibody conjugates of various types, accounting for approximately 14% of the total. Given the rapid development of this field, it is believed that the proportion of these drugs that are approved will increase in the future.

**Table 2:** ADCs first approved or in late-stage clinical trials in oncology^b^

INN^a^	Drug Code(s)	Target	Payload	First global approval or in late-stage clinical trials^c^
Brentuximab vedotin	Adcetris	CD30	MMAE	US, 2011
Ado-trastuzumab emtansine	Kadcyla	HER-2	DM1	US, 2012
Inotuzumab ozogamicin	BESPONSA	CD22	Calicheamicin	US, 2017
Gemtuzumab ozogamicin	Mylotarg	CD33	Calicheamicin	US, 2000
Polatuzumab vedotin	Polivy	CD79β	MMAE	US, 2019
Enfortumab vedotin	Padcev	Nectin-4	MMAE	US, 2019
[fam-]trastuzumab deruxtecan	Enhertu	HER-2	DXd	US, 2019
Sacituzumab govitecan	TRODELVY	TROP-2	SN38	US, 2020
Belantamab mafodotin	BLENREP	BCMA	MMAF	US, 2020
Loncastuximab tesirine	Zynlonta	CD19	PBD dimer SG3199	US, 2021
Tisotumab vedotin	TIVDAK	Tissue factor	MMAE	US, 2021
Mirvetuximab soravtansine	(Pending)	Folate receptor alpha	DM4	In review
Disitamab vedotin	Aidixi	HER-2	MMAE	China, 2021
Zilovertamab vedotin	MK-2140	ROR1	MMAE	Phase II/III (NCT05139017)
Upifitamab rilsodotin	XMT-1536	NaPi2b	Auristatin F-hydroxypropylamide	Phase III (NCT05329545)
Tusamitamab ravtansine	SAR408701	CEACAM5	DM4	Phase III (NCT04154956)
Telisotuzumab vedotin	ABBV-399	cMet	MMAE	Phase III (NCT04928846)
Patritumab deruxtecan	U3-1402	HER3	DXd/DX-8951	Phase III (NCT05338970)
Datopotamab deruxtecan	DS-1062	TROP-2	deruxtecan DX-8951	Phase III (NCT04656652; NCT05555732; NCT05104866)
Camidanlumab tesirine	ADCT-301	CD25	pyrrolobenzodiazepine dimer SG3249	Phase II (pivotal) (NCT04052997)
-	ARX788	HER2	Amberstatin 269 (Auroxime)	Phase II (pivotal), Phase II/III, Phase III (NCT04829604, ACE-Breast-02 (China)) (ACE-Gastric-02 (China))
-	SKB264	Trop-2	Belotecan-derived payload	Phase III pending (NCT05347134)
Pivekimab sunirine	IMGN632	CD123	DGN549 IGN	Phase II (pivotal) (NCT03386513)
Trastuzumab rezetecan	SHR-A1811	HER2	Rezetecan	Phase III (NCT05424835)
Vobramitamab duocarmazine	MGC018	B7-H3	Duocarmycin	Phase II/III pending (NCT05551117)

^a^Some antibodies have not been found INN (International Non-Proprietary Name)

^b^Table data based on publicly available The Antibody Society (www.antibodysociety.org/antibody-therapeutics-product-data/.) and ClinicalTrials.gov (https://clinicaltrials.gov/)

^c^NCT number: ClinicalTrials.gov identifier

An ADC is made up of three main components: a mAb, a cytotoxic payload, and an appropriate linker. Monoclonal antibodies are used as “missiles” to target the surface of tumor cells with antigen-specific expression, and then through receptor-mediated endocytosis, cytotoxic small-molecule drugs are released into cells, killing tumor cells [78]. The “missiles” capacity of antibodies is dependent on two factors: target cell surface antigen expression level and the degree of internalization of the target antigen after binding to the ADC. The physicochemical properties of toxins directly affect the killing ability of ADCs on target cells. The early antitumor agents that were linked to mAbs were methotrexate, vinblastine, and doxorubicin [79]. Generally, ADC payloads can be divided into two major categories: (1) the tubulin polymerization inhibitors (maytansinoid and auristatin) [80], including a derivative of maytansine 1 (DM1) [81], monomethyl auristatin E (MMAE) [82], and monomethyl auristatin F (MMAF) [83]. Among them, MMAE is the most mature in the application. (2) DNA-damaging agents (including calicheamicin [84], SN-38 [85], DXd [86], and PBD [87]). DNA topoisomerase Ӏ inhibitors (represented by DXd) are the most promising. Other small-molecule payloads, such as Pseudomonas exotoxin A (PE38) [88] and RNA polymerase II inhibitor (α‐amanitin) are also under investigation [89, 90].

The FDA approved the first ADC drug, a conjugate of an anti-CD33 monoclonal antibody and calicheamicin, Gemtuzumab ozogamicin (GO), for the treatment of patients aged > 60 years with relapsed CD33-positive acute myeloid leukemia (AML) in 2000 [91–93]. Postmarket clinical trials with GO failed to demonstrate improvements in response rate (RR), overall survival (OS), or disease-free survival; instead, treatment-related mortality increased [94]. On September 17, 2017, the FDA approved the use of GO for patients newly diagnosed with CD33-positive acute lymphoblastic leukemia (ALL) and those who had relapsed or refractory CD33-positive AML based on results from three clinical trials (ALFA-0701 [95], AML-19 [96], and MyloFrance-1 [97]). Brentuximab vedotin is an anti-CD30 ADC conjugated with MMAE via a protease-cleavable dipeptide linker [98]. The FDA approved it for use in the treatment of anaplastic large cell lymphoma (ALCL) and relapsed or refractory Hodgkin’s lymphoma (HL) in 2011 [99–101]. Trastuzumab emtansine (T-DM1), an anti-HER-2 ADC drug comprising trastuzumab linked to the tubulin polymerization inhibitor DM1, was the first ADC used for solid tumors. The FDA approved it for use in the treatment of HER-2-positive metastatic breast cancer (mBC) in 2013, as a second-line drug [102–104]. Inotuzumab ozogamicin, comprising a humanized anti-CD22 IgG4 antibody with calicheamicin [105], was also approved for use by the FDA in 2017 for the treatment of relapsed or refractory B-cell precursor ALL [106, 107]. Polatuzumab vedotin (Polivy) is an ADC drug covalently conjugated a monoclonal anti-CD79 β antibody to MMAE. In June 2019, the US FDA granted accelerated approval for the use of polatuzumab vedotin, in combination with bendamustine plus rituximab, for the treatment of adults with relapsed/refractory DLBCL [108]. Enfortumab vedotin-ejfv (EV) was the first approved biologic that specifically targets Nectin-4 as a directed antibody to deliver cytotoxic MMAE-conjugated payload [109] and previously for treating advanced urothelial carcinoma [110].

Currently, the development of a new drug linker system focusing on different types of drugs has been progressing in many clinical trials. DS-8201a, an HER-2-targeting ADC drug, showed potent antitumor activity, structurally comprising a humanized anti-HER-2 monoclonal antibody, and a novel cytotoxic small molecule drug topoisomerase I inhibitor (Dxd) [111–113]. It is being developed for the treatment of HER-2-expressing solid tumors, including non-small cell lung cancer [114], breast cancer [115], gastric cancer [116], and colorectal cancer [117]. It has been under accelerated approval for the treatment of adult patients with unresectable or metastatic HER-2-positive breast cancer in 2020 [118]. Immunomedics is developing the Trop-2-directed antibody sacituzumab govitecan (Trodelvy) due to its ability to inhibit topoisomerase I, which makes it a potential treatment for breast cancer. A new accelerated approval was granted by the FDA in April 2020 for the treatment of adult patients with metastatic triple-negative breast cancer (mTNBC) who have had at least two previous treatments [119–121]. Belantamab mafodotin is an ADC targeting BCMA that was developed for the treatment of relapsed or refractory multiple myeloma by GlaxoSmithKline [122] in the USA and European Union (EU) in August 2020, as a first-in-class drug [123]. Loncastuximab tesirine, is an ADC drug developed for the treatment of B-cell lymphomas targeting CD19. Mantle-cell lymphoma, follicular lymphoma, and acute lymphoblastic leukemia are also being developed treatments. It is currently approved for the treatment of relapsed/refractory DLBCL in the US [124, 125]. On September 20, 2021, the FDA granted accelerated approval for the use of tisotumab vedotin (Tivdak) for the treatment of adult patients with recurrent or metastatic cervical cancer who experienced disease progression during or after chemotherapy [126].

## Bispecific antibodies (BsAbs)

Another current hot spot in the field of antibody drugs is the development of bispecific or multispecific antibodies. As of July 1, 2022, a total of 9 bispecific antibodies were approved for treatment of diseases (Table 3). The bispecific antibody (BsAb) is a molecule designed to recognize two different antigens or two different binding epitopes for the same antigen. There are many types of BsAbs, including both tiny proteins with only two antigen-binding fragments and large molecules resembling IgG with additional domains [127]. Compared with traditional antibodies, bispecific antibodies have an additional specific antigen binding site, so they are more specific, more accurate in targeting tumor cells, and reduce off-target toxicity. BsAbs have the special function of connecting bridges between cells or proteins, which can recruit more immune cells to target tumor cells or inhibit multiple cross-linking of disease targets. They can also conjugate cytotoxic payload, as bsAb-drug conjugates, for better therapeutic effect [128].

**Table 3:** BsAbs first approved or in late-stage clinical trials^b^

Therapeutic Area	INN^a^	Drug Code(s)^a^	Target	First global approval or in late-stage clinical trials^c^
Oncology	Catumaxomab	Removab	EPCAM, CD3	EU, 2009
Oncology	Blinatumomab	Blincyto	CD19, CD3	US, 2014
Oncology	Amivantamab	RYBREVANT	EGFR, cMET	US, 2021
Oncology	Teclistamab	(Pending)	BCMA, CD3	In review
Oncology	Mosunetuzumab	Lunsumio	CD20, CD3	EU, 2022
Oncology	Cadonilimab	-	PD-1, CTLA4	China, 2022
Oncology	Zanidatamab	ZW25	HER2, HER2 (biparatopic)	Phase II (pivotal) and Phase III (NCT04466891) (NCT05152147)
Oncology	Tebotelimab	MGD013	PD-1, LAG-3	Phase II/III (NCT04082364)
Oncology	Talquetamab	JNJ-64407564	GPCR5D, CD3	Phase II (pivotal), Phase III pending (NCT04634552; NCT05455320)
Oncology	Retlirafusp alfa	SHR-1701	PD-L1, TGF-β	Phase II/III; Phase III (NCT04856787; NCT04950322; NCT05132413)
Oncology	Odronextamab	REGN1979	CD20, CD3	Phase II (potentially pivotal) (NCT03888105)
Oncology	Navicixizumab	OMP-305B83	DLL4, VEGF	Phase III pending (NCT05043402)
Oncology	Linvoseltamab	REGN5458	BCMA, CD3	Phase II (potentially pivotal) (NCT03761108)
Oncology	Izalontamab	SI-B001	EGFR, HER3	Phase II/III (NCT05020457; NCT05020769)
Oncology	Ivonescimab	AK112	PD-1, VEGF	Phase III (NCT05184712)
Oncology	Erfonrilimab	KN046	PD-L1, CTLA4	Phase III (NCT04474119; NCT05001724)
Oncology	Epcoritamab	GEN3013	CD20, CD3	Phase III (NCT04628494)
Oncology	Elranatamab	PF-06863135	BCMA, CD3	Phase III (NCT05020236)
Oncology	Bintrafusp alfa	M7824, MSB0011359C	PD-L1, TGFβ	Phase II/III and Phase III (NCT04066491; NCT03631706)
Oncology	Anbenitamab	KN026	HER2, HER2 (biparatopic)	Phase II (pivotal); Phase II/III pending (NCT04521179; NCT04165993; NCT03925974)
Oncology	-	AFM13	CD30, CD16A	Phase II (pivotal) (NCT04101331)
Oncology	-	CTX-009, ES104, ABL001	VEGF-A, DLL4	Phase II/III pending (NCT05506943)
Hematological Disorders	Emicizumab	Hemlibra	Factor Ixa, X	US, 2017
Ophthalmology	Faricimab	Vabysmo	VEGF-A, Ang-2	US, 2022
Neuromuscular disorders	Gefurulimab	ALXN1720	Complement 5, albumin	Phase III pending (NCT05556096)
Cardiovascular/ hemostasis	-	NNC0365-3769	FIXa, FX	Phase III (NCT05053139)

^a^Some antibodies have not been found INN (International Non-Proprietary Name) or Drug Code

^b^Table data based on publicly available The Antibody Society (www.antibodysociety.org/antibody-therapeutics-product-data/.) and ClinicalTrials.gov (https://clinicaltrials.gov/)

^c^NCT number: ClinicalTrials.gov identifier

There are two major formats of BsAbs: IgG-like formats (e.g., catumaxomab), and non-IgG-like formats (e.g., blinatumomab). The IgG-like BsAbs formats bring about Fc domain-mediated functions, such as ADCC, CDC, and ADCP. The IgG-like BsAbs formats also preserve the physical property of the Fc domain, improve molecular stability and prolong half-life, and maintain serum stability. However, non-IgG-like formats such as bispecific T-cell engagers (BiTEs) lack the entire Fc region. BiTEs composed of two distinct single-chain variable fragments (scFvs) covalently linking CD3 and tumor-associated antigens (TAAs) via small linker peptides are small (~ 55 kDa) and highly flexible, also have pharmacokinetic implications that may avoid toxicity associated with Fc receptor-mediated effector functions, but has a short half-life [129].

The early therapeutic BsAbs were BiTEs, and T-cell activation was first determined in the mid-1980s [130, 131]. BiTE antibody constructs enable the simultaneous binding of CD3ζ within the TCR complex to cell-surface TAAs for MHC-independent TAA targeting specifically to kill the tumor cells [132]. In 2014, blinatumomab, a BiTE targeting CD3/CD19, was first approved for the treatment of hematological complete remission patients with B-cell acute lymphoblastic leukemia (B-ALL) [133, 134]. Catumaxomab (Removab) was approved initially as a T-cell-engaging trifunctional BsAb for the treatment of malignant ascites in 2009. It targets epithelial cell adhesion molecule (EpCAM), which is widely expressed by abdominal tumors, and its other Fab arm recognizes a specific CD3 molecular target on T cells and binds to CD32A by utilizing an engineered Fc arm [135]. However, in 2013, it was spontaneously taken off the market for commercial reasons. Currently, catumaxomab is being conducted for treating patients with peritoneal metastatic gastric cancer in a phase III clinical trial in China (NCT04222114). Tebentafusp-tebn (Kimmtrak), is a bispecific gp100/CD3 T-cell redirection for the treatment of metastatic uveal melanoma using bentafusp [136, 137].

A developing number of BsAbs have been used to treat cancer target immune checkpoint molecules or oncogenic signaling pathways and cytokines, or tumor-associated antigens have been approved for use or are in late-phase clinical trial development. Erfonrilimab (PD-L1/CTLA-4 BsAb) and cadonilimab (PD-1/CTLA-4 BsAb) are both in late-phase clinical trial in China. There is ongoing research with Erfonrilimab in the KN046 trials for the treatment of metastatic NSCLC and advanced pancreatic ductal adenocarcinoma [138, 139]; aside from that, it has also been designated an orphan drug (ODD) for treating thymic epithelial tumors by the FDA. Clinical trials are being conducted for cadonilimab treatment of gastric and gastroesophageal junction adenocarcinomas, as well as cervical cancer [140, 141], and have been granted fast-track designation and ODD by the FDA in China [142]. PD1/VEGF-A BsAbs, ivonescimab, are also being evaluated for making use of chemotherapy to treat EGFR + metastatic NSCLC patients who failed treatments with EGFR inhibitors in a phase III trial [143]. Tebotelimab, a dual affinity retargeting molecule targeting PD-1 and LAG-3, combines with margetuximab and chemotherapy to treat HER-2^+^ gastric/GOJ cancer [144, 145].

**Fig. 4:**
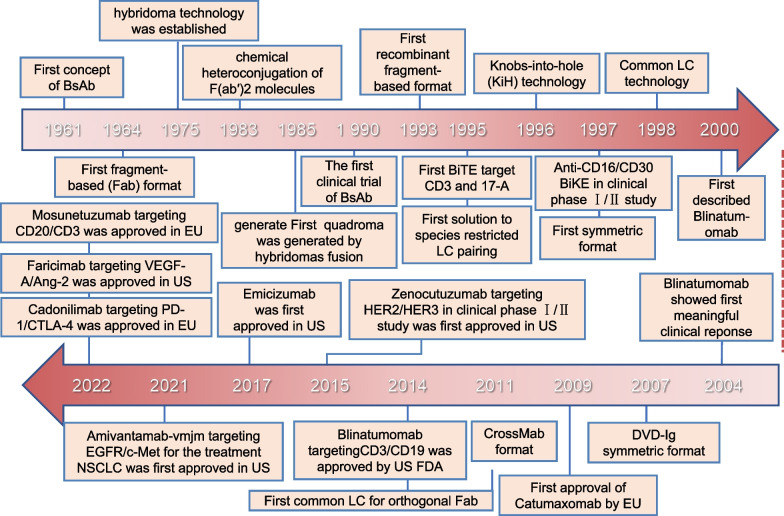
Timeline of historical bispecific antibody developments and first approval for market. This timeline demonstrates key points in the development of bispecific antibodies. BiKEs, bispecific killer engagers; DVD-Ig, dual-variable-domain immunoglobulin

Emicizumab-kxwh (Hemlibra) is a bispecific humanized anti-factor IXa/factor X monoclonal antibody with an Fc region [146]. Its mechanism involves imitating the functions of coagulation factor VIII; after combining with coagulation factors IXa and X, it promotes the degradation of factor X into factor Xa and releases factor Xa so that the coagulation cascade can be carried out and completed in patients with hemophilia A [147, 148]. Amivantamab-vmjm (Rybrevant), targeting EGFR and the mesenchymal-epithelial transition factor (c-Met) mutant signaling pathway, was first approved for the treatment of NSCLC on 21 May 2021 in the USA [149, 150]. Faricimab-svoa (Vabysmo), both targeting VEGF-A and angiopoietin-2 (Ang-2), is being developed for use in the treatment of retinal vascular diseases in experiments that began in January 2022 by Roche/Genentech [151]. Navicixizumab simultaneously inhibits the activity of VEGF and DLL4, which are involved in proangiogenic Notch signaling, and has received fast-track designation from the FDA for the treatment of platinum-resistant ovarian cancer [152, 153]. SI-B001, targeting EGFR and HER-3, has been reported to treat patients with malignancies that are dependent on EGFR and HER-3 heterodimerizerization and that activate downstream oncogenic AKT signaling [154, 155]. Zanidatamab is an HER-2/HER-2 bsAb of two nonoverlapping antigen-binding epitopes. An ongoing phase III trial is testing the combination of zanidatamab with chemotherapy with or without tislelizumab. The trial is testing both treatments for cancers that are HER-2 positive as well as cancers of the gastric/gastroesophageal junction [156] (Fig. 4).

## Antibody fragments (Fab, scFv, VHH) and analogs

**Fig. 5:**
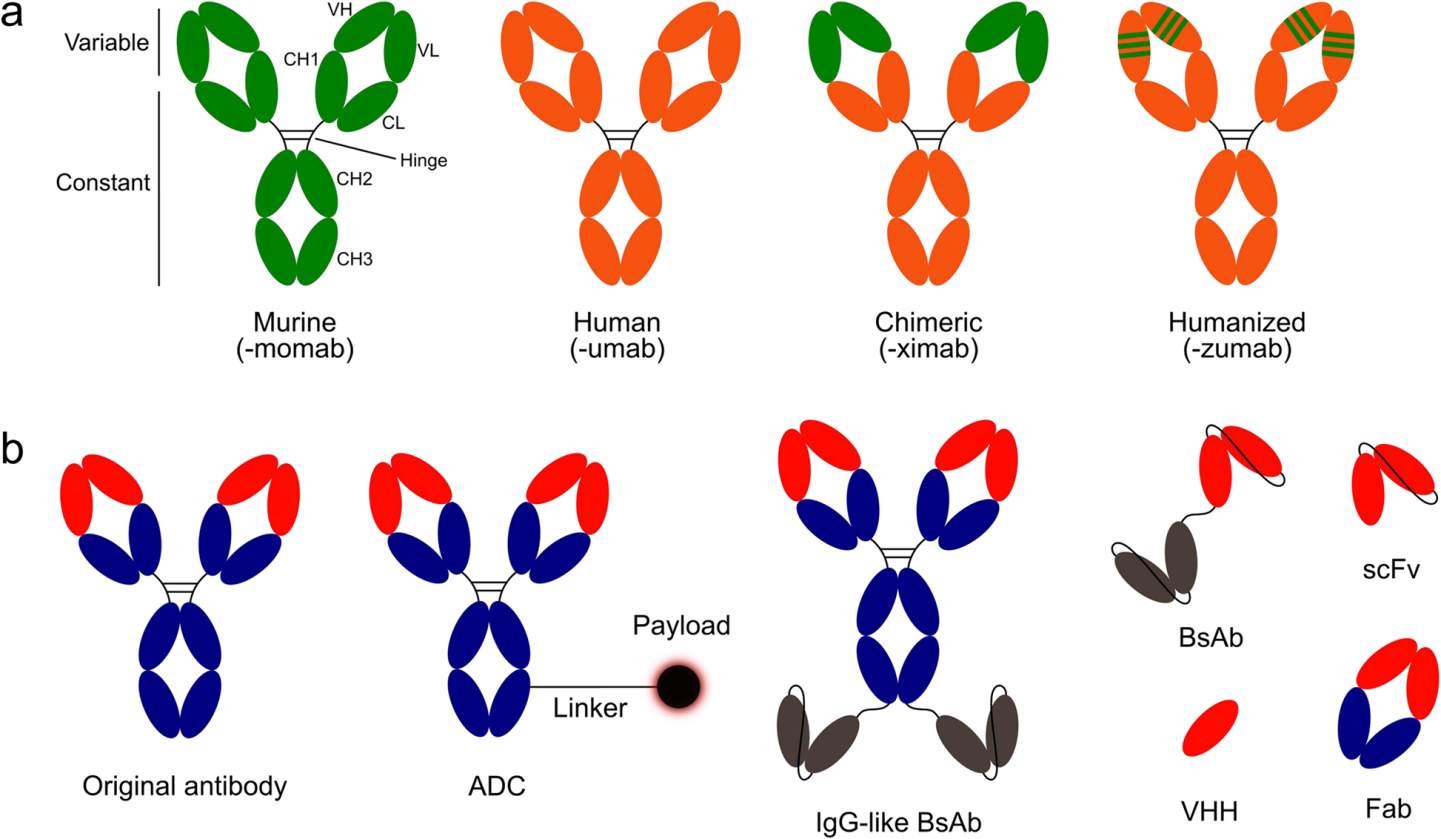
Schematic overview of mAb, ADC, BsAb, and antibody fragments (Fab, scFv, VHH). **a** Antibody humanization from the murine antibody (green domain) to human antibody (orange domain) and associated suffixes. The chimeric mAb: the variable region is of murine origin, and the rest of the chain is of human origin. Humanized mAb: only includes the hypervariable segment of murine origin. CH: domains of the constant region of the heavy chain; CL: constant domain of the light chain; VH: variable domain of the heavy chain; VL: variable domain of the light chain. **b** Original antibody includes variable regions, also called VH and VL (red domain), and the constant region (blue domain). ADC comprises a mAb connected to a cytotoxic payload via an appropriate linker. BsAb consists of two linked antigen-binding fragments (red and gray domains) with two major formats: IgG-like BsAb and non-IgG-like BsAb; Fab consists of the light chain (VL + CL) and the domains of the heavy chain (VH and CH1). scFv is composed of the VH and VL joined by a short flexible polypeptide linker. VHH only contains one heavy chain variable region

In addition to whole antibodies, researchers also use antibody fragments [157], such as Fab [158, 159], scFv [160], and VHH [161], during drug development. As of July 1, 2022, a total of 11 antibody fragments were approved for treatment of diseases (Supplementary Table 2). ScFv is a molecule composed of the VH and VL of an antibody under the link of a peptide chain. Additionally, according to the binding and structural characteristics of the antigen, different antibody analogs have been developed, such as Anticalin [162, 163], Centyrin [164], designed ankyrin repeat proteins (DARPins) [165], Affibody [166] and Knottin [167]. Centyrins (~ 10 kDa), based on the type-III fibronectin (Fn3) domain of tenascin C scaffolds with multiple loops, is similar to the CDR regions of IgGs and impart target specificity [168]. DARPins include b-turns followed by two antiparallel α-helices based on ankyrin repeats [169]. Since they are robust and extremely stable, they can be developed into a multitude of more advanced formats and applications than antibodies and exhibit the same specificities and affinities as antibodies [170]. Affibody is derived from Staphylococcus protein-A Z-domains [171], and are small (~ 7 kDa), 3-helix bundle proteins that contain no cysteine proteins [172]. The affibody ABY-025 was engineered to bind HER-2 with low picomolar affinity via phage display and affinity maturation [173, 174]. Then ABY-025 was subsequently optimized the scaffold region to provide improved hydrophilicity and thermal and chemical stability [175]. Knottins are small (~ 3–6 kDa) with 30–50 amino acid residues in length and exhibit excellent biological, chemical, and thermal stability [176–178]; they are the most prominent antibodies and their analogs currently available for research. Unlike whole antibodies, antibody fragments or antibody analogs have the advantages of a small molecular weight, intense penetration, and low production cost. As a result, they have gained increasing popularity in therapeutic antibody fields. In particular, VHH, a single-domain antibody fragment, also known as a nanobody, has been isolated from immunized camelids [179, 180]. At present, VHH is the most popular antibody fragment in research and development, and it is also the variety that has been approved for use or entered the clinic. Caplacizumab (Cablivi) [181], a humanized VHH targeting Von Willebrand factor, was first approved an EUA for the treatment of acquired thrombotic thrombocytopenic purpura (aTTP) in conjunction with plasma exchange and immunosuppression in the EU in 2018, and then was approved by the US FDA in 2019 [182]. It is undergoing priority review for the treatment of patients aged ≥ 18 years experiencing an episode of aTTP in the USA [183, 184]. Envafolimab (KN035) developed by Corning Jereh, was created by the fusion of the anti-PD-L1 nanobody with the Fc fragment of a human IgG1 antibody [185, 186]. It was approved for the treatment of adult patients with deficient mismatch repair (dMMR) or previously treated microsatellite instability-high (MSI-H) advanced solid tumors in China [187, 188]. In addition, many Chinese and foreign teams are developing many new inhaled drug products of VHH antibodies for the treatment and prevention of SARS-CoV-2. On February 28, 2022, the U.S. FDA approved ciltacabtagene autoleucel (CARVYKTI) for the treatment of adults with relapsed or refractory multiple myeloma (RRMM) [189–191] (Fig. 5).

## Therapeutic antibodies for COVID-19 interventions

Currently, a pandemic of unprecedented scale has been engulfing the world as a result of COVID-19, which caused SARS-CoV-2. COVID-19 requires urgent therapeutic and prophylactic interventions. In the absence of SARS-CoV-2 vaccines, antiviral monoclonal antibodies offer an appealing alternative to neutralizing viruses as soon as they can be isolated and fabricated [192]. After the monoclonal neutralizing antibody binds to the virus, it can promote phagocytosis and elimination of the virus by macrophages. Additionally, monoclonal antibodies can neutralize infected cells, which promotes phagocytosis by macrophages, activates antibodies, and complement-dependent apoptosis, to accelerate the clearance of infected cells.

As the COVID-19 pandemic began in early 2020, there are more than a hundred antibody therapeutics had already been granted approval or emergency use authorization (EUA) or were in clinical studies for COVID-19 interventions, or such authorizations had been requested [193]. As a result of the rapid increase in this number in less than two years, we chose the COVID-19 disease as a representative for analyzing therapeutic antibodies against it. We list therapeutic mAbs which have been granted approval or EUA or in late-stage clinical trials for COVID-19 (Table 4).

**Table 4:** MAbs first approved or in late-stage clinical trials for COVID-19^b^

INN, code or Drug Code(s)^a^	Target	First global approval or in late-stage clinic trials	COVID-19 indication(s)^c^
Bebtelovimab, LY-3853113, LY-CoV 1404	SARS-CoV-2	US, 2022	IV treatment of mild to moderate COVID-19 [194]
Romlusevimab, BRII-198	SARS-CoV-2	China, 2021	Treatment for adults hospitalised with COVID-19 [195]
Cilgavimab/Tixagevimab, Evusheld, AZD-7442	SARS-CoV-2	US, 2021	Pre-exposure prophylaxis of COVID-19 [196]
Regdanvimab, Regkirona	SARS-CoV-2	Republic of Korea, 2021	Mild symptoms of COVID-19 in adult patients [197]
Sotrovimab, XEVUDY	SARS-CoV-2	Australia, 2021	Mild-to-moderate COVID-19 [198]
Casirivimab/Imdevimab, REGEN-COV2	SARS-CoV-2	Japan, 2021	Treatment and prevention of COVID-19 [199]
Levilimab	IL6RA	Russia, 2020	Treatment for severe COVID-19 pneumonia [200]
Tocilizumab, Ilsira	IL6RA	Japan, 2021	Mechanically ventilated patients with COVID-19 [201]
Lananelumab	KLKB1	Phase III	Treatment for hospitalized with COVID-19 (NCT04590586)
Canakinumab	TNFα	Phase III	COVID-19 pneumonia patients (NCT04348448)
Adalimumab	TNFα	Phase III	mild-moderate COVID-19 (NCT04705844)
Leronlimab	CCR5	Phase III	COVID-19 pneumonia (NCT04901676)
Lenzilumab, KB-003	CSF2	Phase III	COVID-19 pneumonia (NCT04351152)
Olokizumab	IL-6	Phase III	COVID-19 pneumonia (NCT05187793)
TY-027	SARS-CoV-2	Phase III	Acutely infected COVID-19 patients (NCT04649515)
Bamlanivimab, LY CoV 555	SARS-CoV-2	Phase III	Mild or Moderate COVID-19 (NCT05205759)
Lomtegovimab, BI-00767551	SARS-CoV-2	Phase II/III	mild to moderate COVID-19 (NCT04822701)
Vilobelimab, CaCP-29	C5	Phase II/III	Severe COVID-19 pneumonia (NCT04333420)
CT-P63	SARS-CoV-2	Phase III	COVID-19 pneumonia (NCT05224856)
Upanovimab, SCTA-01	SARS-CoV-2	Phase II/III	Hospitalized patients with severe COVID-19 (NCT04644185)
INM005	SARS-CoV-2	Phase III	Moderate to severe COVID-19 (NCT04494984)
Pamrevlumab, FG-3019	CCN2	Phase III	COVID-19 pneumonia (NCT05262309)
CPI-006	NT5E	Phase III	mild to moderately symptomatic hospitalized Covid-19 (NCT04734873)
MAD-0004J08	SARS-CoV-2	Phase II/III	Asymptomatic to moderately severe COVID-19 (NCT04952805)
Plonmarlimab	GM-CSF2	Phase III	Severe COVID-19 (NCT04341116)
Adintrevimab (ADG20)	SARS-CoV-2	Phase II/III	Prevention of COVID-19 (NCT04859517)
XAV-19	SARS-CoV-2	Phase II/III	moderate-to-severe COVID-19 (NCT04928430)

^a^Some antibodies have not been found INN (International Non-Proprietary Name) or Drug Code

^b^Table data based on publicly available The Antibody Society (www.antibodysociety.org/antibody-therapeutics-product-data/.) and ClinicalTrials.gov (https://clinicaltrials.gov/)

^c^NCT number: ClinicalTrials.gov identifier

Antibodies against SARS-CoV-2 have been authorized for emergency use in several countries. For example, bebtelovimab, a fully human IgG1 mAb targeting the receptor binding domain (RBD), has been assessed for use during the ongoing pandemic [202]. It has been granted an FDA EUA for the treatment of mild to moderate COVID-19 in patients with intravenous (IV) [194]. A European Union Authorization for Omicron subvariants was authorized by FDA on February 11, 2022. Romlusevimab (BRII-198), a human IgG1 antibody developed by Brii Biosciences that targets distinct epitopes of the SARS-CoV-2 spike protein, was first approved for use on December 8, 2021, in China. AZD7442 (cilgavimab + tixagevimab), which was first isolated from convalescent patients after SARS-CoV-2 infection, a combination of two humans have been reported that AZD7442 could neutralize SARS-CoV-2 variants, including Alpha (B.1.1.7), Beta (B.1.351), Gamma (P.1), Delta (B.1617, AY.1, AY.2, AY.3), or Iota (B.1526) in vitro [203].

It is worth noting that full approval has also been requested or has already been granted for the use of sotrovimab in Australia, but its EUA for treatment of COVID-19 was revoked by FDA on April 5, 2022, due to it is no longer authorized to treat COVID-19 in any U.S. region caused by the Omicron BA.2 sub-variant [204]. The first SARS-CoV-2-specific mAb to be used for COVID-19 therapy began on May 28, 2020, when bamlanivimab was started in a clinical trial on hospitalized COVID-19 patients. November 9, 2020, the FDA granted a EUA for bamlanivimab to be administered as a single infusion to adults and children with mild to moderate COVID-19. Due to the E484 mutation, however, it does not provide protection against Beta (B.1.351), Gamma (P.1), Delta (B.1617, AY.1, AY.2, AY.3) variants [205–208]. Based on emerging data, bamlanivimab alone is not effective against common variants of SARS-CoV-2, so the FDA revoked bamlanivimab's EUA on April 9, 2021. The FDA issued an emergency use authorization for bamlanivimab and etesevimab, both unapproved products, for treating mild to moderate COVID-19 in adults and pediatric patients, including neonates, who have a positive SARS-CoV-2 viral test and who are at high risk of developing severe COVID-19, including hospitalization or death. However, Focosi D, et al. report simultaneous resistance to bamlanivimab and etesevimab via in vivo selection of a SARS-CoV-2 spike mutation (Q493R) [209]. After receiving its first approval in Japan in July 2021 for the treatment of mild to moderate COVID-19 in July 2021, REGEN-COV (casirivimab/imdevimab) received conditional approval in the UK in August 2021 [199, 210, 211]. It was announced by the FDA on January 24, 2022, that two monoclonal antibodies, REGEN-COV and bamlanivimab and etesevimab (administered together), could only be used when a patient has been exposed to or infected with a variant that is susceptible to these therapies [212, 213]. Differently from casirivimab/imdevimab and sotrovimab, the European Medicines Agency (EMA) has never recommended authorizing the combination bamlanivimab/etesevimab for treating COVID-19 (NCT05205759). However, it gave EUA to tixagevimab/cilgavimab, casirivimab/imdevimab, and bamlanivimab/etesevimab when used in combination for to prevent SARS-CoV-2 transmission [214].

## Engineering discovery strategies for therapeutic mAbs

**Fig. 6:**
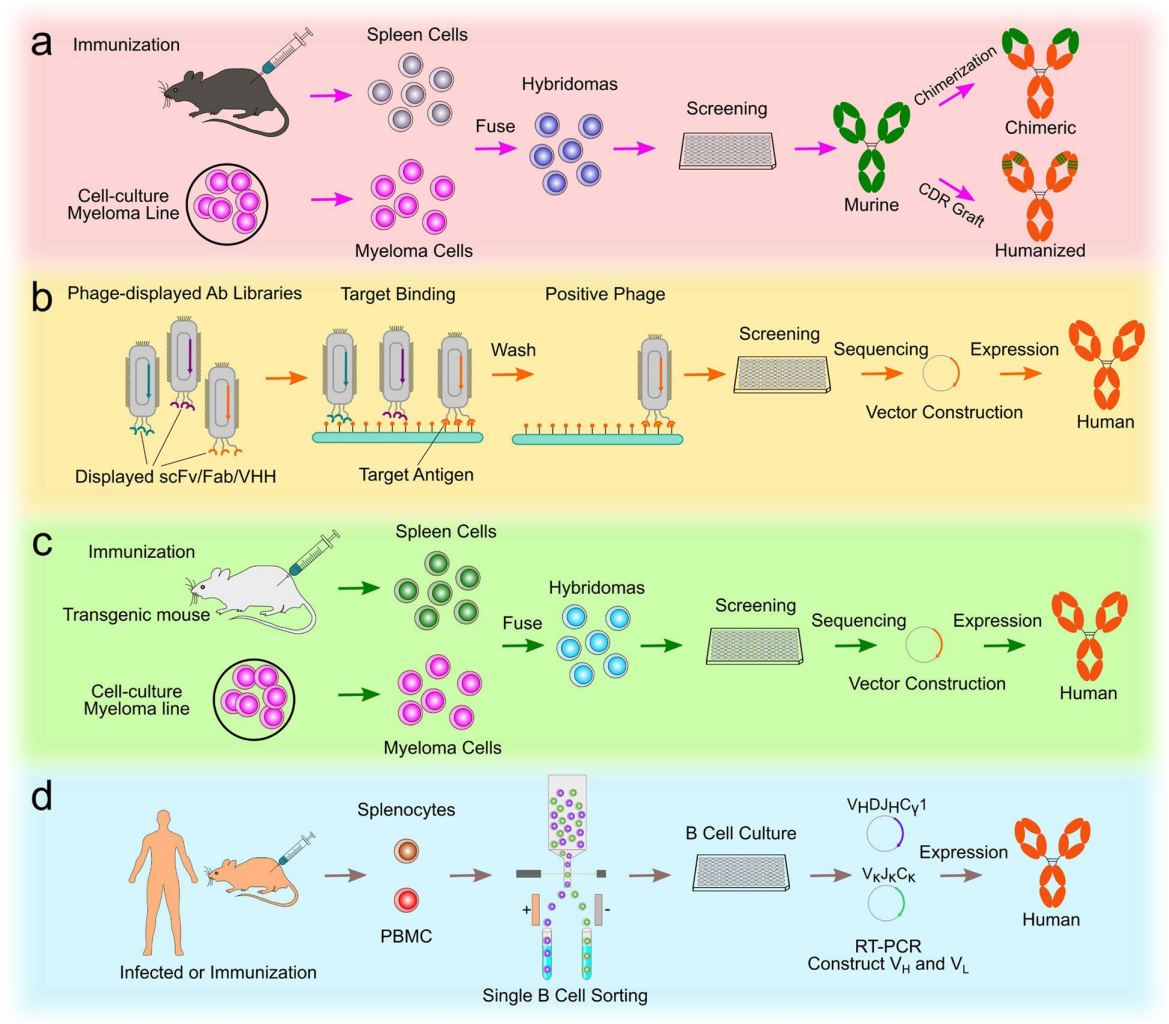
Technologies for the development of therapeutic antibodies. **a** Mouse hybridoma technique. Mice are immunized with desired antigens to induce high immune titers. Myeloma cells and harvested splenocytes are fused to produce hybridomas that persist in secreting antibodies. A chimeric or humanized antibody is then created after the screening has been completed. **b** Phage display. A library of human antibodies is constructed and fused to the gene that encodes the pIII coat protein on the surface of the phage. After binding the target antigen of bio-panning, positive phage clones are screened and then DNA sequences are analyzed to construct and express human IgG. **c** Transgenic mouse. The mice are genetically engineered to contain one or more human immunoglobulin loci which are capable of undergoing gene rearrangement and gene conversion in the transgenic mice to produce diversified human immunoglobulins. Then the fully human antibody screen approach is similar to the mouse hybridoma technique. **d** The single B-cell technique. From infected or immunization donors, PBMCs are prepared for the isolation of suitable B cells by flow cytometry. Following the RT-PCR, VH and VL information of each B cell informs the generation of human mAbs

Antibody engineering discovery is the only way to develop monoclonal antibody drugs, bispecific antibody drugs, ADC drugs, CAR-T, and other cell therapy drugs. Various antibody preparation methods, such as antibody libraries, humanized mice, single B cells, AI-assisted design, and others, have been developed since the invention of monoclonal antibodies based on hybridoma technology in 1975. To date, mouse hybridoma technology is still the most widely and successfully used approach for producing mouse mAbs. Most of the approved therapeutic antibodies are derived from hybridoma mice. It is theoretically possible to screen and select therapeutic antibodies for any target from any organism, thanks to the expansion of antibody discovery techniques such as phage display antibody library, transgenic animals, and human a single B-cell technology (Fig. 6). A transformational impact on antibody discovery and engineering is possible with artificial intelligence and machine learning, though it is still largely unrealized.

### Hybridoma technology

In 1975, Georges JF. Kohler and Milstein successfully manufactured B lymphocyte and myeloma cell fusion cells (hybridoma), which can be cultured in vitro. These cells can proliferate indefinitely and secrete monoclonal antibodies, enabling the application of monoclonal antibodies. Because lymphocytes from different animal species can be used to establish myeloma cells for hybridoma fusion screening, researchers have been able to routinely prepare hybridomas from mice, rats, hamsters, and rabbits. However, hybridomas also have obvious disadvantages, such as a cumbersome preparation process and a long overall preparation cycle. Additionally, mouse immunoglobulin has been found to cause severe host immune responses, which define the HAMA response [215]. As mice antibodies are immunogenic, they can cause rapid clearance, diminished efficacy, and adverse reactions during intravenous infusions in humans [216], and the symptoms include mild fevers, rashes, and cardiopulmonary and anaphylactic reactions [217].

Some researchers have observed higher titers in a short period and have shortened the immunization cycle by improving the adjuvant composition and immune forms. Dempsey et al. found that the complement of C3d can act as a molecular adjuvant to bridge innate and acquired immunity [218]. A study by Chang, J et al. reported that there has been extensive testing of incomplete Freund's adjuvant (IFA) in humans, which demonstrated increased antibody production in comparison to other alum adjuvants, and good tolerance [219]. Subsequent studies in BALB/c mice demonstrated that synthetic oligodeoxynucleotides containing immunostimulatory CpG motifs (CpG ODNs) were powerful adjuvants to proteins given intramuscular injection (IM) or intranasal inhalation (IN) [220, 221]. Garg, R. et al. developed a three components composition adjuvant platform (TriAdj), namely, a TLR agonist, either poly (I:C) or CpG ODNs, and host defense peptide or polyphosphazene [222]. The composition adjuvant platform has been tested on mice, cotton rats, sheep, pigs, and koalas and shows high stability and effectiveness. Common forms of immunization, such as transcutaneous immunization [223], tail vein immunization [224], foot pad immunization [225], and the topical application of antigens and adjuvants can safely and effectively elicit systemic immune responses in mice against a variety of antigens. In recent years, different from traditional protein immunization, next-generation DNA Immunization has been shown to be an efficient approach for immunogen design [226]. Wang, S. et al. compared the relative immunogenicity of a DNA vaccine administered by IM injection, gene gun (GG), or electroporation (EP) to produce antibodies, and the results indicated that both the GG and EP methods were more immunogenic than the IM method [227].

Regarding the HAMA response, Biopharmaceutical companies have addressed this problem using homology sequence alignment, CDR grafting, and surface amino acid modification to generate less immunogenic antibody molecules. Antibody variable regions are not completely variable and can generally be divided into relatively conserved FR and truly variable regions (CDR). The framework amino acid residues of mouse antibodies were replaced with corresponding human amino acid residues in vitro. Only the CDR regions of murine antibodies are grafted to the human antibody framework and constant areas, resulting in humanized antibodies. Likewise, when selecting humanized antibody framework region templates, researchers prefer to use human germline sequences or consensus sequences as a source of templates, rather than selecting those in which the frame sequence may have high-frequency somatic mutations [228–231]. If certain amino acids in the FR framework of a murine antibody are critical for maintaining antigen recognition, these residues need to be preserved. On this basis, in order to further reduce the potential immunogenicity of murine residues on antibody CDR sequences, researchers have invented an antibody humanization technique called specificity-determining residue (SDR) grafting [232]. This method does not require the transplantation of the entire murine CDR into the human framework; instead, only the SDR in the CDR sequence necessary for antigen-binding activity is transplanted into the human framework [233]. By analyzing mutational changes in antibody-combining sites, or by evaluating antigen–antibody complexes with known structures, the SDR can be identified [234]. Thus, the potential immunogenicity risk of antibody variable regions is also minimized. There are also many online databases and software for antibody humanization, which include the Tabhu [235], BioPhi [236], Hu-mAb [237] and IMGT database [238]. If the affinity of the antibody is greatly altered after humanization, it is also necessary to perform back mutation to return to the key amino acid sites of the mouse antibody sequence. Of the most predominant antibody types, approximately 50% are humanized antibodies.

However, the biggest disadvantage of humanized antibody technology is the lack of general methods. Humanization of each molecule requires case analysis, molecular modeling, extensive modification, and trial and error. In addition, due to the presence of murine sequences, the use of humanized monoclonal antibodies cannot completely prevent the risk of immune rejection or hypersensitivity.

### Phage display human antibody libraries

As a result of advanced design technologies such as human antibody phage display libraries, fully humanized antibodies are all encoded by human antibody genes, reducing the heterologous protein content of mouse-derived antibodies. Phage display technology is a very important research method in the field of drug discovery. At present, the FDA has approved many peptide and antibody drugs derived from this technology for the treatment of different diseases, including immune thrombocytopenic purpura, hereditary angioedema, uveitis, and rheumatoid arthritis.

Phage display technology was first used in 1985 to express cloned antigens on a viral surface [239]. A foreign protein, such as an antibody, retains its ability to fold properly and bind to antigens while fusing to the pIII coat protein gene of phage and displaying on its surface [240]. This technology has contributed greatly to the development of therapeutic antibodies used in clinics and was the first technique developed for antibody display screening. Professors George P. Smith and Gregory P. Winter won the Nobel Prize in Chemistry for their pioneering work on “phage display of peptides and antibodies” technology in 2018 [241]. Gradually, it has been demonstrated successfully over time to identify potent, fully human mAbs using these phage-displayed antibody libraries [242].

Currently, the commonly used display formats mainly involve antibody fragment phage display libraries, including the Fab library [243, 244], scFv library [245, 246], and VHH library [247], etc. It is possible to convert intact IgG antibodies from antibody Fab fragments displayed on phage coat proteins with a very small loss of binding activity [248, 249]. High-affinity binders can be retrieved more efficiently from camelid immune VHH libraries. Unlike humans, camelids have only heavy chain antibodies in their blood, which lack the light chain. According to the gene source from which the antibody or antibody fragment is obtained, it can be classified into a natural library [240], an immune library [11], or a synthetic library [250, 251].

Reverse transcription of PBMCs' mRNA into cDNA produces high-quality cDNA to obtain VH and VL gene sequences. Phagemid vectors expressing VH and VL PCR products are ligated into the open reading frame (ORF) region of the pIII protein gene. E. coli harboring a phagemid are infected an M13 helper phage to display functional antibody fragments fused to pIII protein. The phagemid lacks all other genes that encode bacteriophages except the gene for pIII and the origin replication. Virus particles must be assembled using wild-type coat proteins provided by the helper phage, in order to supplement these missing genes [252]. It is called “panning” when antibodies are selected in vitro from libraries. It takes several steps to complete the affinity enrichment process. In these steps, the target antigen is immobilized, phages are bound to the target antigen, unbound phages are eliminated, and phages are eluted [253–255].

### Human antibodies from transgenic mice

These biological macromolecules have inherent immunogenicity that has influenced the development of rodent antibodies as therapeutics. With the advancement of gene editing technology, using human antibody transgenic mice to produce humanized antibodies is no longer a dream. The development of therapeutic antibodies is made possible by transgenic animals. There are several advantages to producing antibodies from transgenic animals over other technologies. These include the lack of humanization, the increase in diversity, the ability to essentially mature antibodies in vivo, and the ability to optimize antibodies through clonal selection. However, developing transgenic mouse antibody technology proved challenging due to the large size of human Ig genes. It is also necessary to express the human variable (V), diversity (D), and joining (J) segments in high amounts in transgenic mice. This will enable us to produce repertoires similar or comparable to those in humans [256]. In order to overcome these difficulties, researchers have created fully human antibody mice and chimeric human antibody mice to express human antibody libraries in transgenic animals.

In 1985, transgenic mice were first proposed as a means to produce fully human antibodies. Alt et al. suggest that unrearranged germline-configuration transgenes could be converted into new human sequence mAbs using transgenic technology [257]. Even though this approach seemed outlandish on the surface, the authors said it could “be realized shortly”. In 1989, Brüggemann M. et al. [258] created transgenic mice carrying a human heavy-chain minilocus comprising unrearranged immunoglobulin V, D, and J segments linked to a human mu-chain (C_μ_) gene. A study reported that in an unrearranged germline configuration, the light chain and heavy chain minilocus transgenes containing human immunoglobulin coding sequences were expressed to produce a diverse repertoire of CDR3s, and these miniloci were characterized, as well as the CDR3 repertoire produced [259].

Additionally, mice deficient in Ig were developed in parallel. Chen, J. et al. reported that after targeting the J_H_ gene segments in embryonic stem cells, they created mice unable to assemble IgH chains in 1993 [260]. In the same year, they knocked out the mouse J_L_ gene again, inactivating mouse Ig [261]. A transgenic human IgH and IgL mouse was bred with an IgH knockout mouse and a mouse lacking IgL to create lines that were able to produce more diverse human antibodies. The first human Ig transgenic mouse strain containing transgenes of human sequences, undergoes V(D)J joining and heavy-chain classes switching, as well as somatic mutation, to produce a repertoire of human sequence immunoglobulins, was developed in 1994 [262]. In 2007, A. Jakobovits et al. used the XenoMouse transgenic system, which successfully recapitulated the human antibody response in mice, with the inactivated mouse antibody machinery [263]. Even though this line replaced mouse endogenous Ig with human Ig genes and eliminated interference from mouse endogenous Ig, human antibody production efficiency, Ig class switching, and somatic hypermutation still remain low because mice do not express the constant region gene. The murine Fc modulates the signaling for somatic hypermutation during antibody affinity maturation and effector function of antibodies on condition that chimeric antibodies can be generated in mice with human Fab and murine Fc region [264, 265]. In 2013, Osborn, M. J. et al. generated A novel transgenic rat line exclusively expressing chimeric Abs with human idiotypes has been generated using a humanized rat strain (OmniRat) carrying chimeric human/rat IgH and human IgL loci [266]. Human V(D)J transcripts were highly diverse, and B-cell recovery was non-discriminatory from wild-type animals. OmniRat strains produced high-affinity serum IgG after immunization as effectively as normal rats. Mouse chimeric antibodies can be produced after antigen immunization through somatic hypermutation.

### Single B-cell antibody technology

Somatic recombination is the primary mechanism for generating a diverse repertoire of B-cells, which is necessary for a robust immune response. The rearrangement of discrete germline gene segments establishes B-cell diversity since the human genome is limited [267, 268]. In Ig-transgenic mouse systems, human immune responses cannot be precisely replicated since the murine genetic background affects antigen processing and B-cell regulation. It is important to note that, despite this drawback, most fully human antibodies are produced today using mouse systems that use the immunoglobulin transgene. Select antibodies can be affinity matured in vitro using display technologies [269, 270]. However, they are usually based on random combinations, which means that VH and VL antibodies typically do not pair naturally with each other. A strategy based on the direct amplification of genes encoded by the VH and VL regions from single human B cells and their subsequent expression in cell culture systems has been designed in order to maintain the original VH and VL pairing that exists in human B cells, which is a method capable of being used to immortalize B cells.

In 1977, Steinitz, M. et al. reported for the first time that Epstein-Barr virus (EBV) could be used to induce the immortalization of B cells that could preserve the characteristics of the original B cell [271]. Since then, many scientists have studied the method of preparing monoclonal antibodies from EBV-immortalized B cells [272–274]. However, it was found that the efficiency of EBV immortalization of B cells is very low, and the concentration of antibodies produced is also minimal, so this method has not been widely used until now.

After the development of flow cytometry screening technology, it was possible to sort out antigen-specific B lymphocytes by using B-cell surface markers, followed by the use of a multicolor flow cytometry instrument. Magnetic microbeads conjugated with B-cell-specific markers, or fluorescence-activated cell sorting based on individual B cells' cell surface markers, are used to isolate individual B cells from human PBMCs [275]. Using single-cell RT-PCR and expression vector cloning, a new platform has been developed for generating genes for the heavy chain and light chain of human B cells [276, 277]. To evaluate the Ig transcriptome repertoires of single B cells, Shi, Z. et al. used Chromium Single-Cell Immune Profiling Solution and Sanger sequencing. In some single B cells, multi-Ig specificity is found at unprecedented levels, revealing that immunoglobulin gene rearrangements and class switching are regulated differently than in classical “single-cell to single-antibody” formation [278]. A number of technologies have emerged in the past two decades to assess the repertoire of B-cells and antibodies at high resolution. A breakthrough microfluidic system called single-cell RNA sequencing has been developed, which encodes transcriptomic data from individual cells [279, 280]. Basically, microfluidics involves encapsulating cells in oil in aqueous droplets and adding a barcoded gel bead. Each barcoded primer captures mRNA for reverse transcription after the cells are lysed. A library preparation is made and sequencing is performed on the cDNAs from all droplets. In post-sequencing analysis, the transcripts derived from each gel bead with their corresponding VH-VL pairings can be reunified by barcoding each gel bead individually. By calculating the clonal diversity within and between patient samples, downstream analyses can be performed in order to determine the intensity of the immune response, as well as quantify the rate of somatic hypermutation (SHM) and the length of hypervariable CDR3 regions within the antibody sequence to determine the extent of affinity maturation [277, 281].

Unlike traditional hybridoma technology, single B cells have many advantages, such as the flow sorting stage, which can be used to effectively enrich antigen-specific B cells and reduce the loss of positive cells due to the cell fusion step. Another obvious advantage is that fully human antibodies can be directly screened from human peripheral blood, which is very suitable during infectious disease research. Especially for acute infectious diseases such as SARS-CoV-2, this technology can be used to rapidly isolate fully human antibodies from the peripheral blood of patients, which can be used in subsequent new drug development. The currently approved SARS-COV-2 neutralizing antibodies are derived from single B-cell cloning technology. For example, Tsinghua University Professor Zhang Linqi's team reported a study of 8 individuals infected with SARS-CoV-2 was conducted to isolate and characterize monoclonal antibodies specific for RBD [282], leading to the approval of the first anti- SARS-CoV-2 antibody in China. Bamlanivimab (LY-CoV555), etesevimab (LY-CoV016) [283–286], and sotrovimab (Xevudy) are all recombinant human monoclonal antibodies targeted against SARS-CoV-2 isolated from a single B-cell clone, respectively [287, 288].

### AI-assisted antibody discovery

In 1972, Christian Anfinsen famously hypothesized the following in his acceptance speech for the Nobel Prize in Chemistry: in theory, a protein amino acid sequence should completely determine its structure. In the following decades, people have been exploring methods to calculate and predict the three-dimensional structure of proteins from their primary amino acid sequences. Due to the rapid development of structural biology techniques, an increasing number of protein three-dimensional structures are being stored in the Protein Data Bank (PDB) database; the rapid development of computer technology and deep learning methods has also occurred. Researchers have finally made breakthroughs in the field of protein structure prediction in recent years. On November 17, 2021, Science magazine announced its 2021 annual list of scientific breakthroughs, which included AlphaFold [289, 290] and RoseTTAfold [291], two technologies for predicting protein structures based on artificial intelligence, at the top of the list [292–294]. For instance, using AlphaFold begins with the sequences and structures of approximately 100,000 known proteins to train the model, and then results in a well-trained model that can be used to predict the shape of a protein, at scale and in minutes, down to atomic accuracy. This work has cross-era importance since researchers have been able to accurately predict the three-dimensional structure of proteins and even the interactions between proteins by means of artificial intelligence. It also lays the foundation for scientists to explore the use of AI technology to design specific binding antibodies, optimize the sequence structure of antibodies that bind to antigens, and screen the potential optimal antibodies that bind to antigens.

The application of AI or deep learning in antibody development mainly includes the following three aspects: (1) Sequence-to-structure prediction. For antibodies, predicting accurately the structure of the CDR, specifically CDR-H3 (Predicting CDR H3 loop structures with geometric potentials from deep learning), remains the major challenge in terms of structure prediction. The use of deep learning-based methods for predicting antibody structures has recently been demonstrated to be more accurate than methods that are trained to predict general structures, like those of other models. For example, Jeffrey A. Ruffolo et al. developed a deep learning-based approach known as DeepAb, which can be used for structure prediction and optimization of antibodies [295]. To generate structures from network predictions, DeepAb uses a Rosetta-based protocol and a deep neural network to predict distances and orientations between residues. (2) Antigen–antibody docking, interaction prediction, and affinity maturation. For example, Constantin Schneider et al. recently developed a deep learning-based software structure-based deep learning for antibodies (DLAB). Antibodies with no known antibodies bind to antigen targets of interest or can be predicted to bind against antigen targets of interest using this method [296]. Brennan Abanades et al. presented ABlooper, which provides a confidence estimate for each prediction of the CDR loop structure using end-to-end equivariant deep learning based on the CDR loop structure [297]. (3) Prediction of antigen epitope. For example, Rahmad Akbar et el. reported that they identified some antigen–antibody interaction motifs through the analysis of a large number of existing antigen–antibody interactions. These motifs will be conducive to the further development of deep learning-based antibody-antigen interaction prediction and epitope prediction [298]. Additionally, antibody sequences are designed using generative machine learning techniques beyond predictive applications. Deep learning also has applications in antibody humanization, such as the BioPhi software developed by David Prihoda, which can be used for antibody design and humanization analysis [236].

There are an increasing number of reports combining artificial intelligence for antibody optimization. Derek M. Mason reported on deep learning techniques for predicting antigenic specificity of antibody sequences in order to optimize therapeutic antibodies [299]. Sisi Shan et al. introduced an antibody that broadly neutralized SARS-CoV-2 variants and was identified using a deep learning approach to redesign CDRs to target multiple virus variants [300]. In March 2022, David Baker's team reported in Nature that they were able to design engineered proteins that bind to the target protein based on only the structure of the target protein [301]. An increasing number of studies have shown that artificial intelligence has broad application prospects in antibody optimization and protein design and engineering.

## Future perspectives

It is possible to divide therapeutic antibodies into two broad categories. In the first category, naked antibodies can be used directly to treat diseases. The development of new technologies has recently contributed to the enhancement of ADCC or CDC therapeutic effects, such as antibody point mutations [302] or glycosyl modifications that lead to improved cancer cell killing capabilities [303–305]. These approaches include optimizing the hinge and crystallizable fragment of antibodies or modifying antibody glycosylation to enhance or reduce antibody effector functions and circulating half-life [306]. Therapeutic antibodies are typically induced to apoptosis directly in cancer cells. In terms of modifying the tumor microenvironment, antibodies are capable of inhibiting tumorigenesis by focusing on factors involved in the growth of cancer cells. For example, Cancer cells cannot grow if they do not have access to nutrients, which VEGF inhibits by inhibiting the growth of blood vessels around them [307]. The mechanism of action of the antibody itself has been unable to effectively cure most malignant tumors. It is also possible that antibody-based therapy for human diseases will be more effective and specific if novel biomarkers and targets are identified.

The second category of antibody drugs consists of antibodies with additional modifications to enhance their therapeutic properties. Some general approaches include antibody–drug conjugates, antibody-radionuclide conjugates, bispecific antibodies, the use of immune cytokines, immunoliposomes, and chimeric antigen receptor T-cell (CAR-T) therapy. Radiation immunotherapy is experiencing a good prospect for development. It is particularly effective because it uses antibodies for specific targeting and small molecular radioisotope warheads for rapid pharmacokinetics. Thus, they are particularly powerful in treating heterogeneous cancers [308]. To ensure the efficacy of ADCs, high antigen expression and high toxicity payload are required. ADC drugs gain new ideas with the development of bispecific technology. One antibody binds to tumor cell-associated antigen, and the other antibody promotes the endocytosis and degradation of the molecule, enhancing the efficacy of ADC drugs when faced with targets with weaker internalization [309]. Proteolysis-targeting chimeras (PROTAC) is a drug development technology that utilizes the ubiquitin–proteasome system (UPS) to degrade target proteins. This heterobifunctional molecule can specifically degrade the target protein in cells. Clinically, PROTAC have additional advantages over pure target inhibition [310, 311]. By connecting a chemical linker to PROTAC payloads, degradation-antibody conjugates act as a new class of antibody drugs. The currently validated degradation-antibody conjugates show in vitro or in vivo biological activity, which will be a new mode of conjugation with clinical significance [312].

Currently, X-ray crystallography, cryo-electron microscopy, and computer-assisted homology modeling can be used to analyze the structure of antigen–antibody complexes and elucidate an understanding of the key residues that act directly to guide antibody humanization modification. Many web service platforms can provide informatics and antibody structure databases, which provide technical support for human antibody framework region selection, antibody modeling, etc. Multiple tools for quantifying the degree of monoclonal antibody humanization, such as the H⁃score, G-score, and T20 analysis designed by Abhinandan and Martin [313], can also be used to clearly separate human sequences from mouse sequences and those of other species. It is expected that derived antibody drugs will be multi-mechanistic and multifunctional, and that the design of antibody drugs will encompass biological, chemical, mathematical and computer science fields more broadly and deeply.

## Conclusions

After decades of development, therapeutic antibodies have played an increasingly important role in the treatment of malignant tumors, autoimmune diseases, infections, and other diseases, and are currently one of the focuses and hot spots of international innovative drug development. This article summarizes the types of therapeutic antibodies, the marketed and underdevelopment antibodies, and the application status of five antibody development technologies: hybridomas, antibody libraries, humanized mice, single B cells, and artificial intelligence. In general, the preparation technologies used to generate antibodies, including mature hybridoma technology, emerging single B-cell technology, and antibody library technology that does not depend on antigen immunization, are developing rapidly. The antibody humanization process is omitted in humanized mouse technology, and deep learning algorithms assist in antibody humanization and antibody virtual screening design. Researchers can flexibly adopt suitable screening methods according to the specific requirements of the project. At the same time, pure antibody drugs are also developing in the direction of antibody-conjugated drugs, bispecific antibodies, and multispecific antibodies, and new varieties are constantly entering the clinic or being approved. Looking to the future, with the rapid development of theories and technologies in all aspects of antibodies, the development of therapeutic antibodies will become increasingly mature, and the speed from target discovery to antibody drugs entering the clinic will also become faster. However, regarding the marketed and underresearched antibody varieties, the problems of concentrated targets, a high degree of homogeneity, excessive competition, and waste of social and clinical resources are apparent. In the future, only by focusing on clinical needs, meeting the unmet needs of patients, and continuing to increase investment in basic research, such as research on disease mechanisms and new target discovery, can the adequate development of the antibody–drug market be better promoted.

## Supplementary Information

**Additional file 1: Supplementary Table 1.** Monoclonal antibodies in late-stage clinical trials^a^.

**Additional file 2: Supplementary Table 2.** Antibody fragments first approved or in late-stage clinical trials^a^.

## Data Availability

Not applicable.

## Abbreviations

VEGF: Vascular endothelial growth factor
CTLA-4: Cytotoxic t-lymphocyte antigen 4
LAG-3: Lymphocyte-activation gene 3
HER-2: Epidermal growth factor receptor 2
EGFR: Epidermal growth factor receptor
DDL4: Delta-like ligand 4
HER-3: Epidermal growth factor receptor 3
IGF-1R: Insulin like growth factor-1 receptor
BLyS: B Lymphocyte stimulator
IFNAR1: Interferon alpha and beta receptor subunit 1
EpCAM: Epithelial cell adhesion molecule
VEGFR2: Vascular endothelial growth factor receptor 2
SLAMF7: Signaling lymphocytic activation molecule family member 7
PDGFRα: Platelet-derived growth factor receptor alpha
CCR4: CC chemokine receptor 4
TROP-2: Trophoblast cell-surface antigen 2
BCMA: B cell maturation antigen
B7-H3: B7 homolog 3
RSV: Respiratory syncytial virus
SARS-CoV-2: Severe acute respiratory syndrome coronavirus 2
GPIIb/IIIa: Glycoprotein IIb/IIIa
MASP-2: MBL associated serine protease 2
CGRP: Calcitonin gene-related peptide
FGF23: Fibroblast growth factor 23
RANK-L: Receptor activator for nuclear factor-κB ligand
PCSK9: Proprotein convertase subtilisin/kexin type 9
ROR1: Receptor tyrosine kinase like orphan receptor 1
TIGIT: T cell immunoglobulin and ITIM domain
NaPi2b: The sodium-dependent phosphate transporter
CEACAM5: Carcinoembryonic antigen-related cell adhesion molecule 5
GPCR5D: The orphan G protein-coupled receptor, class C group 5 member D
TIM-3: T cell immunoglobulin and mucin domain 3
TGF-β: Transforming growth factor-β
CA125: Cancer antigen 125
OX40: The tumour necrosis factor receptor
FGFR2b: The fibroblast growth factor receptor 2
PSMA: The pennsylvania septage management association
APRIL: A proliferation-inducing ligand
GM-CSF: Granulocyte–macrophage colony stimulating factor
KLKB1: Kallikrein B1
NT5E: The ecto-5-prime-nucleotidase

## References

[1] Köhler G, Milstein C (1975). Continuous cultures of fused cells secreting antibody of predefined specificity. Nature.

[2] Schwaber J (1982). Hybridoma technology. Science.

[3] Meeker TC, Lowder J, Maloney DG, Miller RA, Thielemans K, Warnke R (1985). A clinical trial of anti-idiotype therapy for B cell malignancy. Blood.

[4] Richards JM, Vogelzang NJ, Bluestone JA (1990). Neurotoxicity after treatment with muromonab-CD3. N Engl J Med.

[5] Faulds D, Sorkin EM (1994). Abciximab (c7E3 Fab). A review of its pharmacology and therapeutic potential in ischaemic heart disease. Drugs..

[6] Jones PT, Dear PH, Foote J, Neuberger MS, Winter G (1986). Replacing the complementarity-determining regions in a human antibody with those from a mouse. Nature.

[7] Tsurushita N, Hinton PR, Kumar S (2005). Design of humanized antibodies: from anti-Tac to Zenapax. Methods.

[8] Vincenti F, Kirkman R, Light S, Bumgardner G, Pescovitz M, Halloran P (1998). Interleukin-2-receptor blockade with daclizumab to prevent acute rejection in renal transplantation. N Engl J Med.

[9] Ferrara N, Hillan KJ, Gerber H-P, Novotny W (2004). Discovery and development of bevacizumab, an anti-VEGF antibody for treating cancer. Nat Rev Drug Discov.

[10] McCafferty J, Griffiths AD, Winter G, Chiswell DJ (1990). Phage antibodies: filamentous phage displaying antibody variable domains. Nature..

[11] Clackson T, Hoogenboom HR, Griffiths AD, Winter G (1991). Making antibody fragments using phage display libraries. Nature.

[12] Kempeni J (1999). Preliminary results of early clinical trials with the fully human anti-TNFα monoclonal antibody D2E7. Ann Rheum Dis..

[13] Keystone EC, Kavanaugh AF, Sharp JT, Tannenbaum H, Hua Y, Teoh LS (2004). Radiographic, clinical, and functional outcomes of treatment with adalimumab (a human anti-tumor necrosis factor monoclonal antibody) in patients with active rheumatoid arthritis receiving concomitant methotrexate therapy: a randomized, placebo-controlled, 52-week trial. Arthritis Rheum.

[14] Jaffe GJ, Dick AD, Brézin AP, Nguyen QD, Thorne JE, Kestelyn P (2016). Adalimumab in patients with active noninfectious uveitis. N Engl J Med.

[15] Kimball AB, Okun MM, Williams DA, Gottlieb AB, Papp KA, Zouboulis CC (2016). Two phase 3 trials of adalimumab for hidradenitis suppurativa. N Engl J Med.

[16] Menter A, Tyring SK, Gordon K, Kimball AB, Leonardi CL, Langley RG (2008). Adalimumab therapy for moderate to severe psoriasis: a randomized, controlled phase III trial. J Am Acad Dermatol.

[17] Lonberg N (2005). Human antibodies from transgenic animals. Nat Biotechnol.

[18] Gibson TB, Ranganathan A, Grothey A (2006). Randomized phase III trial results of panitumumab, a fully human anti-epidermal growth factor receptor monoclonal antibody, in metastatic colorectal cancer. Clin Colorectal Cancer.

[19] Moroni M, Veronese S, Benvenuti S, Marrapese G, Sartore-Bianchi A, Di Nicolantonio F (2005). Gene copy number for epidermal growth factor receptor (EGFR) and clinical response to anti-EGFR treatment in colorectal cancer: a cohort study. Lancet Oncol.

[20] Ferris RL, Blumenschein G, Fayette J, Guigay J, Colevas AD, Licitra L (2016). Nivolumab for recurrent squamous-cell carcinoma of the head and neck. N Engl J Med.

[21] Riaz N, Havel JJ, Makarov V, Desrichard A, Urba WJ, Sims JS (2017). Tumor and microenvironment evolution during immunotherapy with nivolumab. Cell.

[22] Mullard A (2021). FDA approves 100th monoclonal antibody product. Nat Rev Drug Discov.

[23] Hafeez U, Parakh S, Gan HK, Scott AM (2020). Antibody-drug conjugates for cancer therapy. Molecules..

[24] Brinkmann U, Kontermann RE (2021). Bispecific antibodies. Science.

[25] Kallewaard NL, Corti D, Collins PJ, Neu U, McAuliffe JM, Benjamin E (2016). Structure and function analysis of an antibody recognizing all influenza a subtypes. Cell.

[26] Stanfield RL, Wilson IA. Antibody structure. Microbiol Spectr. 2014;2(2). 10.1128/microbiolspec.AID-0012-2013 10.1128/microbiolspec.AID-0012-2013 26105818

[27] Sun Y, Izadi S, Callahan M, Deperalta G, Wecksler AT (2021). Antibody-receptor interactions mediate antibody-dependent cellular cytotoxicity. J Biol Chem.

[28] Tay MZ, Wiehe K, Pollara J (2019). Antibody-dependent cellular phagocytosis in antiviral immune responses. Front Immunol.

[29] Oostindie SC, van der Horst HJ, Kil LP, Strumane K, Overdijk MB, van den Brink EN (2020). DuoHexaBody-CD37(®), a novel biparatopic CD37 antibody with enhanced Fc-mediated hexamerization as a potential therapy for B-cell malignancies. Blood Cancer J.

[30] Liu R, Li W, Meng Y, Gao S, Zhang J, Hu X (2021). Phase I study of pucotenlimab (HX008), an anti-PD-1 antibody, for patients with advanced solid tumors. Ther Adv Med Oncol.

[31] Tolcher AW, Sznol M, Hu-Lieskovan S, Papadopoulos KP, Patnaik A, Rasco DW (2017). Phase Ib study of utomilumab (PF-05082566), a 4–1BB/CD137 agonist, in combination with pembrolizumab (MK-3475) in patients with advanced solid tumors. Clin Cancer Res.

[32] Patnaik A, Kang SP, Rasco D, Papadopoulos KP, Elassaiss-Schaap J, Beeram M (2015). Phase I study of pembrolizumab (MK-3475; anti-PD-1 monoclonal antibody) in patients with advanced solid tumors. Clin Cancer Res.

[33] Rizvi NA, Hellmann MD, Snyder A, Kvistborg P, Makarov V, Havel JJ (2015). Cancer immunology. Mutational landscape determines sensitivity to PD-1 blockade in non-small cell lung cancer. Science..

[34] Garon EB, Rizvi NA, Hui R, Leighl N, Balmanoukian AS, Eder JP (2015). Pembrolizumab for the treatment of non-small-cell lung cancer. N Engl J Med.

[35] Reck M, Rodríguez-Abreu D, Robinson AG, Hui R, Csőszi T, Fülöp A (2016). Pembrolizumab versus chemotherapy for PD-L1-positive non-small-cell lung cancer. N Engl J Med.

[36] Schmid P, Cortes J, Pusztai L, McArthur H, Kümmel S, Bergh J (2020). Pembrolizumab for early triple-negative nreast cancer. N Engl J Med.

[37] Adusumilli PS, Zauderer MG, Rivière I, Solomon SB, Rusch VW, O'Cearbhaill RE (2021). A phase I trial of regional mesothelin-targeted CAR T-cell therapy in patients with malignant pleural disease, in combination with the anti-PD-1 agent pembrolizumab. Cancer Discov.

[38] Motzer RJ, Escudier B, McDermott DF, George S, Hammers HJ, Srinivas S (2015). Nivolumab versus everolimus in advanced renal-cell carcinoma. N Engl J Med.

[39] Borghaei H, Paz-Ares L, Horn L, Spigel DR, Steins M, Ready NE (2015). Nivolumab versus docetaxel in advanced nonsquamous non-mall-cell lung cancer. N Engl J Med.

[40] Chowdhury S, Bappy MH, Clocchiatti-Tuozzo S, Cheeti S, Chowdhury S, Patel V (2021). Current advances in immunotherapy for glioblastoma multiforme and future prospects. Cureus.

[41] Morgensztern D, Herbst RS (2016). Nivolumab and pembrolizumab for non-small cell lung cancer. Clin Cancer Res.

[42] Croxtall JD (2011). Ustekinumab. Drugs.

[43] Sandborn WJ, Feagan BG, Fedorak RN, Scherl E, Fleisher MR, Katz S (2008). A randomized trial of ustekinumab, a human interleukin-12/23 monoclonal antibody, in patients with moderate-to-severe crohn's disease. Gastroenterology.

[44] Feagan BG, Sandborn WJ, Gasink C, Jacobstein D, Lang Y, Friedman JR (2016). Ustekinumab as induction and maintenance therapy for crohn’s disease. N Engl J Med.

[45] Sands BE, Sandborn WJ, Panaccione R, O’Brien CD, Zhang H, Johanns J (2019). Ustekinumab as induction and maintenance therapy for ulcerative colitis. N Engl J Med.

[46] Plosker GL, Figgitt DP (2003). Rituximab: a review of its use in non-Hodgkin's lymphoma and chronic lymphocytic leukaemia. Drugs.

[47] Smith MR (2003). Rituximab (monoclonal anti-CD20 antibody): mechanisms of action and resistance. Oncogene.

[48] Urbain R, Teillaud JL, Prost JF (2009). Les anticorps EMABling(R): De la prophylaxie de l'allo-immunisation foeto-maternelle au traitement de la leucémie lymphoïde chronique [EMABling antibodies: from feto-maternal allo-immunisation prophylaxis to chronic lymphocytic leukaemia therapy]. Med Sci (Paris).

[49] Sharman JP, Farber CM, Mahadevan D, Schreeder MT, Brooks HD, Kolibaba KS (2017). Ublituximab (TG-1101), a novel glycoengineered anti-CD20 antibody, in combination with ibrutinib is safe and highly active in patients with relapsed and/or refractory chronic lymphocytic leukaemia: results of a phase 2 trial. Br J Haematol.

[50] Fox E, Lovett-Racke AE, Gormley M, Liu Y, Petracca M, Cocozza S (2021). A phase 2 multicenter study of ublituximab, a novel glycoengineered anti-CD20 monoclonal antibody, in patients with relapsing forms of multiple sclerosis. Mult Scler.

[51] Babiker HM, Glode AE, Cooke LS, Mahadevan D (2018). Ublituximab for the treatment of CD20 positive B-cell malignancies. Expert Opin Investig Drugs.

[52] Liossis SN, Staveri C (2021). What's new in the treatment of systemic lupus erythematosus. Front Med (Lausanne).

[53] Szili D, Cserhalmi M, Bankó Z, Nagy G, Szymkowski DE, Sármay G (2014). Suppression of innate and adaptive B cell activation pathways by antibody coengagement of FcγRIIb and CD19. MAbs.

[54] Chu SY, Pong E, Bonzon C, Yu N, Jacob CO, Chalmers SA (2021). Inhibition of B cell activation following in vivo co-engagement of B cell antigen receptor and Fcγ receptor IIb in non-autoimmune-prone and SLE-prone mice. J Transl Autoimmun.

[55] Mora J, Chan GC-F, Morgenstern DA, Nysom K, Bear MK, Dalby LW (2020). Naxitamab, a new generation anti-GD2 monoclonal antibody (mAb) for treatment of relapsed/refractory high-risk neuroblastoma (HR-NB). J Clin Oncol.

[56] Mora J, Castañeda A, Gorostegui M, Santa-María V, Garraus M, Muñoz JP (2021). Naxitamab combined with granulocyte-macrophage colony-stimulating factor as consolidation for high-risk neuroblastoma patients in complete remission. Pediatr Blood Cancer.

[57] Fu Y, Yu J, Liatsou I, Du Y, Josefsson A, Nedrow JR (2022). Anti-GD2 antibody for radiopharmaceutical imaging of osteosarcoma. Eur J Nucl Med Mol Imaging.

[58] Slatnick LR, Jimeno A, Gore L, Macy ME (2021). Naxitamab: a humanized anti-glycolipid disialoganglioside (anti-GD2) monoclonal antibody for treatment of neuroblastoma. Drugs Today (Barc).

[59] Markham A (2021). Naxitamab: first approval. Drugs.

[60] Frampton JE (2021). Isatuximab: a review of its use in multiple myeloma. Target Oncol.

[61] Martin T, Strickland S, Glenn M, Charpentier E, Guillemin H, Hsu K (2019). Phase I trial of isatuximab monotherapy in the treatment of refractory multiple myeloma. Blood Cancer J..

[62] Mikhael J, Belhadj-Merzoug K, Hulin C, Vincent L, Moreau P, Gasparetto C (2021). A phase 2 study of isatuximab monotherapy in patients with multiple myeloma who are refractory to daratumumab. Blood Cancer J.

[63] Bang LM, Keating GM (2004). Adalimumab. BioDrugs.

[64] Held J, Esse J, Tascilar K, Steininger P, Schober K, Irrgang P (2021). Reactogenicity correlates only weakly with humoral immunogenicity after COVID-19 vaccination with BNT162b2 mRNA (Comirnaty®). Vaccines (Basel).

[65] Faro-Viana J, Bergman M-L, Gonçalves LA, Duarte N, Coutinho TP, Borges PC (2022). Population homogeneity for the antibody response to COVID-19 BNT162b2/Comirnaty vaccine is only reached after the second dose across all adult age ranges. Nat Commun..

[66] Sernicola A, Dybala A, Gomes V, Maddalena P, Adotti F, Soda G (2022). Lymphomatoid drug reaction developed after BNT162b2 (Comirnaty) COVID-19 vaccine manifesting as pityriasis lichenoides et varioliformis acuta-like eruption. J Eur Acad Dermatol Venereol.

[67] Khoja L, Butler MO, Kang SP, Ebbinghaus S, Joshua AM (2015). Pembrolizumab. J Immunother Cancer..

[68] Kwok G, Yau TCC, Chiu JW, Tse E, Kwong Y-L (2016). Pembrolizumab (Keytruda). Hum Vaccin Immunother.

[69] Recondo G, Mezquita L (2022). Tiragolumab and atezolizumab in patients with PD-L1 positive non-small-cell lung cancer. Lancet Oncol.

[70] Cho BC, Abreu DR, Hussein M, Cobo M, Patel AJ, Secen N (2022). Tiragolumab plus atezolizumab versus placebo plus atezolizumab as a first-line treatment for PD-L1-selected non-small-cell lung cancer (CITYSCAPE): primary and follow-up analyses of a randomised, double-blind, phase 2 study. Lancet Oncol.

[71] Goodman KA, Xu R-h, Chau I, Chen MH, Cho BC, Shah MA (2022). SKYSCRAPER-07: A phase III, randomized, double-blind, placebo-controlled study of atezolizumab with or without tiragolumab in patients with unresectable ESCC who have not progressed following definitive concurrent chemoradiotherapy. J Clin Oncol..

[72] Liu J, Gao T, Tan Z, Li S, Xu J, Bai C (2022). Phase II study of TQB2450, a novel PD-L1 antibody, in combination with anlotinib in patients with locally advanced or metastatic soft tissue sarcoma. Clin Cancer Res.

[73] Lan CY, Zhao J, Yang F, Xiong Y, Li R, Huang Y (2022). Anlotinib combined with TQB2450 in patients with platinum-resistant or-refractory ovarian cancer: A multi-center, single-arm, phase 1b trial. Cell Rep Med.

[74] Zhou J, Sun Y, Zhang W, Yuan J, Peng Z, Wang W (2022). Phase Ib study of anlotinib combined with TQB2450 in pretreated advanced biliary tract cancer and biomarker analysis. Hepatology.

[75] Bril V, Benatar M, Andersen H, Vissing J, Brock M, Greve B (2021). Efficacy and safety of rozanolixizumab in moderate to severe generalized myasthenia gravis: a phase 2 randomized control trial. Neurology.

[76] Smith B, Kiessling A, Lledo-Garcia R, Dixon KL, Christodoulou L, Catley MC (2018). Generation and characterization of a high affinity anti-human FcRn antibody, rozanolixizumab, and the effects of different molecular formats on the reduction of plasma IgG concentration. MAbs.

[77] Guttman-Yassky E, Blauvelt A, Eichenfield LF, Paller AS, Armstrong AW, Drew J (2020). Efficacy and safety of lebrikizumab, a high-affinity interleukin 13 inhibitor, in adults with moderate to severe atopic dermatitis: a phase 2b randomized clinical trial. JAMA Dermatol.

[78] Criscitiello C, Morganti S, Curigliano G (2021). Antibody-drug conjugates in solid tumors: a look into novel targets. J Hematol Oncol.

[79] Trail PA, Willner D, Lasch SJ, Henderson AJ, Hofstead S, Casazza AM (1993). Cure of xenografted human carcinomas by BR96-Doxorubicin immunoconjugates. Science.

[80] Kaur R, Kaur G, Gill RK, Soni R, Bariwal J (2014). Recent developments in tubulin polymerization inhibitors: an overview. Eur J Med Chem.

[81] Lambert JM, Chari RVJ (2014). Ado-trastuzumab Emtansine (T-DM1): An antibody-drug conjugate (ADC) for HER2-positive breast cancer. J Med Chem.

[82] Francisco JA, Cerveny CG, Meyer DL, Mixan BJ, Klussman K, Chace DF (2003). cAC10-vcMMAE, an anti-CD30-monomethyl auristatin E conjugate with potent and selective antitumor activity. Blood.

[83] Doronina SO, Mendelsohn BA, Bovee TD, Cerveny CG, Alley SC, Meyer DL (2006). Enhanced activity of monomethylauristatin F through monoclonal antibody delivery: effects of linker technology on efficacy and toxicity. Bioconjug Chem.

[84] Ricart AD (2011). Antibody-drug conjugates of calicheamicin derivative: gemtuzumab ozogamicin and inotuzumab ozogamicin. Clin Cancer Res.

[85] Govindan SV, Cardillo TM, Sharkey RM, Tat F, Gold DV, Goldenberg DM (2013). Milatuzumab-SN-38 conjugates for the treatment of CD74^+^ cancers. Mol Cancer Ther.

[86] Xu Z, Guo D, Jiang Z, Tong R, Jiang P, Bai L (2019). Novel HER2-targeting antibody-drug conjugates of trastuzumab beyond T-DM1 in breast cancer: trastuzumab deruxtecan (DS-8201a) and (Vic-) trastuzumab duocarmazine (SYD985). Eur J Med Chem.

[87] Hartley JA (2021). Antibody-drug conjugates (ADCs) delivering pyrrolobenzodiazepine (PBD) dimers for cancer therapy. Expert Opin Biol Ther.

[88] Onda M, Nagata S, FitzGerald DJ, Beers R, Fisher RJ, Vincent JJ (2006). Characterization of the B cell epitopes associated with a truncated form of <em>pseudomonas</em> exotoxin (PE38) used to make immunotoxins for the treatment of cancer patients. J Immunol.

[89] Pahl A, Lutz C, Hechler T (2018). Amanitins and their development as a payload for antibody-drug conjugates. Drug Discov Today Technol.

[90] Figueroa-Vazquez V, Ko J, Breunig C, Baumann A, Giesen N, Pálfi A (2021). HDP-101, an anti-BCMA antibody-drug conjugate, safely delivers amanitin to induce cell death in proliferating and resting multiple myeloma cells. Mol Cancer Ther.

[91] Bross PF, Beitz J, Chen G, Chen XH, Duffy E, Kieffer L (2001). Approval summary: gemtuzumab ozogamicin in relapsed acute myeloid leukemia. Clin Cancer Res.

[92] Hamann PR, Hinman LM, Hollander I, Beyer CF, Lindh D, Holcomb R (2002). Gemtuzumab ozogamicin, a potent and selective anti-CD33 antibody-calicheamicin conjugate for treatment of acute myeloid leukemia. Bioconjug Chem.

[93] Hamann PR, Hinman LM, Beyer CF, Lindh D, Upeslacis J, Flowers DA (2002). An anti-CD33 antibody-calicheamicin conjugate for treatment of acute myeloid leukemia. Choice of linker. Bioconjug Chem.

[94] Petersdorf SH, Kopecky KJ, Slovak M, Willman C, Nevill T, Brandwein J (2013). A phase 3 study of gemtuzumab ozogamicin during induction and postconsolidation therapy in younger patients with acute myeloid leukemia. Blood.

[95] Castaigne S, Pautas C, Terré C, Raffoux E, Bordessoule D, Bastie J-N (2012). Effect of gemtuzumab ozogamicin on survival of adult patients with de-novo acute myeloid leukaemia (ALFA-0701): a randomised, open-label, phase 3 study. Lancet.

[96] Amadori S, Suciu S, Selleslag D, Aversa F, Gaidano G, Musso M (2016). Gemtuzumab ozogamicin versus best supportive care in older patients with newly diagnosed acute myeloid leukemia unsuitable for intensive chemotherapy: results of the randomized phase III EORTC-GIMEMA AML-19 trial. J Clin Oncol.

[97] Taksin AL, Legrand O, Raffoux E, de Revel T, Thomas X, Contentin N (2007). High efficacy and safety profile of fractionated doses of Mylotarg as induction therapy in patients with relapsed acute myeloblastic leukemia: a prospective study of the alfa group. Leukemia.

[98] Younes A, Bartlett NL, Leonard JP, Kennedy DA, Lynch CM, Sievers EL (2010). Brentuximab vedotin (SGN-35) for relapsed CD30-positive lymphomas. N Engl J Med.

[99] Younes A, Yasothan U, Kirkpatrick P (2012). Brentuximab vedotin. Nat Rev Drug Discov..

[100] Younes A, Gopal AK, Smith SE, Ansell SM, Rosenblatt JD, Savage KJ (2012). Results of a pivotal phase II study of brentuximab vedotin for patients with relapsed or refractory Hodgkin's lymphoma. J Clin Oncol.

[101] Connors JM, Jurczak W, Straus DJ, Ansell SM, Kim WS, Gallamini A (2017). Brentuximab vedotin with chemotherapy for stage III or IV hodgkin’s lymphoma. N Engl J Med.

[102] Zolot RS, Basu S, Million RP (2013). Antibody-drug conjugates. Nat Rev Drug Discov.

[103] Amiri-Kordestani L, Blumenthal GM, Xu QC, Zhang L, Tang SW, Ha L (2014). FDA approval: ado-trastuzumab emtansine for the treatment of patients with HER2-positive metastatic breast cancer. Clin Cancer Res.

[104] Verma S, Miles D, Gianni L, Krop IE, Welslau M, Baselga J (2012). Trastuzumab emtansine for HER2-positive advanced breast cancer. N Engl J Med.

[105] DiJoseph JF, Dougher MM, Armellino DC, Evans DY, Damle NK (2007). Therapeutic potential of CD22-specific antibody-targeted chemotherapy using inotuzumab ozogamicin (CMC-544) for the treatment of acute lymphoblastic leukemia. Leukemia.

[106] Lamb YN (2017). Inotuzumab ozogamicin: first global approval. Drugs.

[107] Kantarjian HM, DeAngelo DJ, Stelljes M, Martinelli G, Liedtke M, Stock W (2016). Inotuzumab ozogamicin versus standard therapy for acute lymphoblastic leukemia. N Engl J Med.

[108] Deeks ED (2019). Polatuzumab Vedotin: first global approval. Drugs.

[109] Chang E, Weinstock C, Zhang L, Charlab R, Dorff SE, Gong Y (2021). FDA approval summary: enfortumab vedotin for locally advanced or metastatic urothelial carcinoma. Clin Cancer Res.

[110] Powles T, Rosenberg JE, Sonpavde GP, Loriot Y, Durán I, Lee J-L (2021). Enfortumab vedotin in previously treated advanced urothelial carcinoma. N Engl J Med.

[111] Ogitani Y, Aida T, Hagihara K, Yamaguchi J, Ishii C, Harada N (2016). DS-8201a, A novel HER2-targeting ADC with a novel DNA topoisomerase I inhibitor, demonstrates a promising antitumor efficacy with differentiation from T-DM1. Clin Cancer Res.

[112] Iwata TN, Ishii C, Ishida S, Ogitani Y, Wada T, Agatsuma T (2018). A HER2-targeting antibody-drug conjugate, trastuzumab deruxtecan (DS-8201a), enhances antitumor immunity in a mouse model. Mol Cancer Ther.

[113] Tamura K, Tsurutani J, Takahashi S, Iwata H, Krop IE, Redfern C (2019). Trastuzumab deruxtecan (DS-8201a) in patients with advanced HER2-positive breast cancer previously treated with trastuzumab emtansine: a dose-expansion, phase 1 study. Lancet Oncol.

[114] Li BT, Smit EF, Goto Y, Nakagawa K, Udagawa H, Mazières J (2021). Trastuzumab deruxtecan in HER2-mutant non-small-cell lung cancer. N Engl J Med.

[115] Modi S, Saura C, Yamashita T, Park YH, Kim S-B, Tamura K (2019). Trastuzumab deruxtecan in previously treated HER2-positive breast cancer. N Engl J Med.

[116] Shitara K, Bang Y-J, Iwasa S, Sugimoto N, Ryu M-H, Sakai D (2020). Trastuzumab deruxtecan in previously treated HER2-positive gastric cancer. N Engl J Med.

[117] Siena S, Sartore-Bianchi A, Marsoni S, Hurwitz HI, McCall SJ, Penault-Llorca F (2018). Targeting the human epidermal growth factor receptor 2 (HER2) oncogene in colorectal cancer. Ann Oncol.

[118] Keam SJ (2020). Trastuzumab deruxtecan: first approval. Drugs.

[119] Syed YY (2020). Sacituzumab govitecan: first approval. Drugs.

[120] Bardia A, Mayer IA, Vahdat LT, Tolaney SM, Isakoff SJ, Diamond JR (2019). Sacituzumab govitecan-hziy in refractory metastatic triple-negative breast cancer. N Engl J Med.

[121] Bardia A, Hurvitz SA, Tolaney SM, Loirat D, Punie K, Oliveira M (2021). Sacituzumab govitecan in metastatic triple-negative breast cancer. N Engl J Med.

[122] Yu B, Jiang T, Liu D (2020). BCMA-targeted immunotherapy for multiple myeloma. J Hematol Oncol.

[123] Markham A (2020). Belantamab mafodotin: first approval. Drugs.

[124] Caimi PF, Ai W, Alderuccio JP, Ardeshna KM, Hamadani M, Hess B (2021). Loncastuximab tesirine in relapsed or refractory diffuse large B-cell lymphoma (LOTIS-2): a multicentre, open-label, single-arm, phase 2 trial. Lancet Oncol.

[125] Lee A (2021). Loncastuximab tesirine: first approval. Drugs.

[126] Markham A (2021). Tisotumab vedotin: first approval. Drugs.

[127] Labrijn AF, Janmaat ML, Reichert JM, Parren P (2019). Bispecific antibodies: a mechanistic review of the pipeline. Nat Rev Drug Discov.

[128] Li H, Er Saw P, Song E (2020). Challenges and strategies for next-generation bispecific antibody-based antitumor therapeutics. Cell Mol Immunol.

[129] Thakur A, Huang M, Lum LG (2018). Bispecific antibody based therapeutics: strengths and challenges. Blood Rev.

[130] Perez P, Hoffman RW, Shaw S, Bluestone JA, Segal DM (1985). Specific targeting of cytotoxic T cells by anti-T3 linked to anti-target cell antibody. Nature.

[131] Staerz UD, Kanagawa O, Bevan MJ (1985). Hybrid antibodies can target sites for attack by T cells. Nature..

[132] Yuraszeck T, Kasichayanula S, Benjamin JE (2017). Translation and clinical development of bispecific T-cell engaging antibodies for cancer treatment. Clin Pharmacol Ther.

[133] Kantarjian H, Stein A, Gökbuget N, Fielding AK, Schuh AC, Ribera J-M (2017). Blinatumomab versus chemotherapy for advanced acute lymphoblastic leukemia. N Engl J Med.

[134] Przepiorka D, Ko C-W, Deisseroth A, Yancey CL, Candau-Chacon R, Chiu H-J (2015). FDA approval: blinatumomab. Clin Cancer Res.

[135] Seimetz D (2011). Novel monoclonal antibodies for cancer treatment: the trifunctional antibody catumaxomab (removab). J Cancer.

[136] Dhillon S (2022). Tebentafusp: first approval. Drugs.

[137] Nathan P, Hassel JC, Rutkowski P, Baurain J-F, Butler MO, Schlaak M (2021). Overall survival benefit with tebentafusp in metastatic uveal melanoma. N Engl J Med.

[138] Zhou C, Xiong A, Li W, Ma Z, Li X, Fang J (2021). P77. 03 A phase II study of KN046 (bispecific anti-PD-L1/CTLA-4) in patients (pts) with metastatic non-small cell lung cancer (NSCLC). J Thoracic Oncol..

[139] Jin G, Guo S, Zhang Y, Ma Y, Guo X, Zhou X (2021). Efficacy and safety of KN046 plus nab-paclitaxel/gemcitabine as first-line treatment for unresectable locally advanced or metastatic pancreatic ductal adenocarcinoma (PDAC). J Clin Oncol.

[140] Ji J, Shen L, Li Z, Xu N, Liu T, Chen Y (2021). AK104 (PD-1/CTLA-4 bispecific) combined with chemotherapy as first-line therapy for advanced gastric (G) or gastroesophageal junction (GEJ) cancer: updated results from a phase Ib study. J Clin Oncol..

[141] Wu X, Wang J, Huang Y, Li Y, Sun Y, Wang K (2022). Abstract 5180: A randomized, double-blind, placebo-controlled phase III study to evaluate AK104 plus platinum-containing chemotherapy with or without bevacizumab as first-line treatment for persistent, recurrent, or metastatic cervical cancer. Cancer Res..

[142] Senior M (2021). China at the threshold. Nat Biotechnol.

[143] Zhao Y, Fang W, Yang Y, Chen J, Zhuang L, Du Y (2022). A phase II study of AK112 (PD-1/VEGF bispecific) in combination with chemotherapy in patients with advanced non-small cell lung cancer. J Clin Oncol..

[144] Catenacci DVT, Rosales MK, Chung HC, Yoon HH, Shen L, Moehler MH (2021). Margetuximab (M) combined with anti-PD-1 (retifanlimab) or anti-PD-1/LAG-3 (tebotelimab) +/- chemotherapy (CTX) in first-line therapy of advanced/metastatic HER2+ gastroesophageal junction (GEJ) or gastric cancer (GC). J Clin Oncol.

[145] Catenacci DVT, Rosales M, Chung HC, H Yoon H, Shen L, Moehler M (2021). MAHOGANY: margetuximab combination in HER2+ unresectable/metastatic gastric/gastroesophageal junction adenocarcinoma. Future Oncol..

[146] Oldenburg J, Mahlangu JN, Kim B, Schmitt C, Callaghan MU, Young G (2017). Emicizumab prophylaxis in hemophilia A with inhibitors. N Engl J Med.

[147] Scott LJ, Kim ES (2018). Emicizumab-kxwh: first global approval. Drugs.

[148] Mahlangu J, Oldenburg J, Paz-Priel I, Negrier C, Niggli M, Mancuso ME (2018). Emicizumab prophylaxis in patients who have hemophilia A without inhibitors. N Engl J Med.

[149] Yun J, Lee S-H, Kim S-Y, Jeong S-Y, Kim J-H, Pyo K-H (2020). Antitumor activity of amivantamab (JNJ-61186372), an EGFR–MET bispecific antibody, in diverse models of EGFR exon 20 insertion-driven NSCLC. Cancer Discov.

[150] Syed YY (2021). Amivantamab: first approval. Drugs.

[151] Shirley M (2022). Faricimab: first approval. Drugs.

[152] Jimeno A, Moore KN, Gordon M, Chugh R, Diamond JR, Aljumaily R (2019). A first-in-human phase 1a study of the bispecific anti-DLL4/anti-VEGF antibody navicixizumab (OMP-305B83) in patients with previously treated solid tumors. Invest New Drugs.

[153] Fu S, Corr BR, Culm-Merdek K, Mockbee C, Youssoufian H, Stagg R, et al. Phase Ib study of navicixizumab plus paclitaxel in patients with platinum-resistant ovarian, primary peritoneal, or fallopian tube cancer. J Clin Oncol. 2022:JCO.21.01801. doi:10.1200/JCO.21.01801 10.1200/JCO.21.01801 PMC9362870 35439029

[154] Haikala HM, Jänne PA (2021). Thirty years of HER3: from basic biology to therapeutic interventions. Clin Cancer Res.

[155] Zhang J, Yi J, Zhou P (2020). Development of bispecific antibodies in China: overview and prospects. Antib Ther.

[156] Lee KW, Im Y-H, Lee KS, Cho JY, Oh D-Y, Chung HCC (2021). Zanidatamab, an anti-HER2 bispecific antibody, plus chemotherapy with/without tislelizumab as first-line treatment for patients with advanced HER2-positive breast cancer or gastric/gastroesophageal junction adenocarcinoma: a phase 1B/2 trial-in-progress. J Clin Oncol..

[157] Holliger P, Hudson PJ (2005). Engineered antibody fragments and the rise of single domains. Nat Biotechnol.

[158] Lu Z-R, Kopečková P, Kopeček J (1999). Polymerizable Fab′ antibody fragments for targeting of anticancer drugs. Nat Biotechnol.

[159] Chen Y, Wiesmann C, Fuh G, Li B, Christinger HW, McKay P (1999). Selection and analysis of an optimized anti-VEGF antibody: crystal structure of an affinity-matured fab in complex with antigen. J Mol Biol.

[160] Chen F, Ma K, Madajewski B, Zhuang L, Zhang L, Rickert K (2018). Ultrasmall targeted nanoparticles with engineered antibody fragments for imaging detection of HER2-overexpressing breast cancer. Nat Commun.

[161] Smolarek D, Hattab C, Hassanzadeh-Ghassabeh G, Cochet S, Gutiérrez C, de Brevern AG (2010). A recombinant dromedary antibody fragment (VHH or nanobody) directed against human Duffy antigen receptor for chemokines. Cell Mol Life Sci.

[162] Peper-Gabriel JK, Pavlidou M, Pattarini L, Morales-Kastresana A, Jaquin TJ, Gallou C (2022). The PD-L1/4-1BB bispecific antibody-anticalin fusion protein PRS-344/S095012 elicits strong T-cell stimulation in a tumor-localized manner. Clin Cancer Res.

[163] Rothe C, Skerra A (2018). Anticalin(®) proteins as therapeutic agents in human diseases. BioDrugs.

[164] Hermanson DL, Barnett BE, Rengarajan S, Codde R, Wang X, Tan Y (2016). A novel bcma-specific, centyrin-based CAR-T product for the treatment of multiple myeloma. Blood.

[165] Stumpp MT, Dawson KM, Binz HK (2020). Beyond antibodies: the DARPin® drug platform. BioDrugs.

[166] Ståhl S, Gräslund T, Eriksson Karlström A, Frejd FY, Nygren P-Å, Löfblom J (2017). Affibody molecules in biotechnological and medical applications. Trends Biotechnol.

[167] Richards DA (2018). Exploring alternative antibody scaffolds: antibody fragments and antibody mimics for targeted drug delivery. Drug Discov Today Technol.

[168] Diem MD, Hyun L, Yi F, Hippensteel R, Kuhar E, Lowenstein C (2014). Selection of high-affinity Centyrin FN3 domains from a simple library diversified at a combination of strand and loop positions. Protein Eng Des Sel.

[169] Guillard S, Kolasinska-Zwierz P, Debreczeni J, Breed J, Zhang J, Bery N (2017). Structural and functional characterization of a DARPin which inhibits Ras nucleotide exchange. Nat Commun..

[170] Plückthun A (2015). Designed Ankyrin Repeat Proteins (DARPins): Binding proteins for research, diagnostics, and therapy. Annu Rev Pharmacol Toxicol.

[171] Nygren PA (2008). Alternative binding proteins: affibody binding proteins developed from a small three-helix bundle scaffold. FEBS J.

[172] Nord K, Gunneriusson E, Ringdahl J, Ståhl S, Uhlén M, Nygren PA (1997). Binding proteins selected from combinatorial libraries of an alpha-helical bacterial receptor domain. Nat Biotechnol.

[173] Sandberg D, Tolmachev V, Velikyan I, Olofsson H, Wennborg A, Feldwisch J (2017). Intra-image referencing for simplified assessment of HER2-expression in breast cancer metastases using the Affibody molecule ABY-025 with PET and SPECT. Eur J Nucl Med Mol Imaging.

[174] Sörensen J, Sandberg D, Sandström M, Wennborg A, Feldwisch J, Tolmachev V (2014). First-in-human molecular imaging of HER2 expression in breast cancer metastases using the 111In-ABY-025 affibody molecule. J Nucl Med.

[175] Feldwisch J, Tolmachev V, Lendel C, Herne N, Sjöberg A, Larsson B (2010). Design of an optimized scaffold for affibody molecules. J Mol Biol.

[176] Kintzing JR, Cochran JR (2016). Engineered knottin peptides as diagnostics, therapeutics, and drug delivery vehicles. Curr Opin Chem Biol.

[177] Kolmar H (2008). Alternative binding proteins: biological activity and therapeutic potential of cystine-knot miniproteins. FEBS J.

[178] Iyer S, Acharya KR (2011). Tying the knot: The cystine signature and molecular-recognition processes of the vascular endothelial growth factor family of angiogenic cytokines. FEBS J.

[179] Zhai T, Wang C, Xu Y, Huang W, Yuan Z, Wang T (2021). Generation of a safe and efficacious llama single-domain antibody fragment (vHH) targeting the membrane-proximal region of 4–1BB for engineering therapeutic bispecific antibodies for cancer. J Immunother Cancer.

[180] Jähnichen S, Blanchetot C, Maussang D, Gonzalez-Pajuelo M, Chow KY, Bosch L (2010). CXCR4 nanobodies (VHH-based single variable domains) potently inhibit chemotaxis and HIV-1 replication and mobilize stem cells. Proc Natl Acad Sci U S A.

[181] Duggan S (2018). Caplacizumab: first global approval. Drugs.

[182] Scully M, Cataland SR, Peyvandi F, Coppo P, Knöbl P, Kremer Hovinga JA (2019). Caplacizumab treatment for acquired thrombotic thrombocytopenic purpura. N Engl J Med.

[183] Peyvandi F, Scully M, Kremer Hovinga JA, Cataland S, Knöbl P, Wu H (2016). Caplacizumab for acquired thrombotic thrombocytopenic purpura. N Engl J Med.

[184] Elverdi T, Eskazan AE (2019). Caplacizumab as an emerging treatment option for acquired thrombotic thrombocytopenic purpura. Drug Des Devel Ther.

[185] Akinleye A, Rasool Z (2019). Immune checkpoint inhibitors of PD-L1 as cancer therapeutics. J Hematol Oncol.

[186] Zhang F, Wei H, Wang X, Bai Y, Wang P, Wu J (2017). Structural basis of a novel PD-L1 nanobody for immune checkpoint blockade. Cell Discov.

[187] Markham A (2022). Envafolimab: first approval. Drugs.

[188] Akinleye A, Rasool Z (2019). Immune checkpoint inhibitors of PD-L1 as cancer therapeutics. J Hematol Oncol.

[189] Van Oekelen O, Aleman A, Upadhyaya B, Schnakenberg S, Madduri D, Gavane S (2021). Neurocognitive and hypokinetic movement disorder with features of parkinsonism after BCMA-targeting CAR-T cell therapy. Nat Med.

[190] Cohen AD, Parekh S, Santomasso BD, Gállego Pérez-Larraya J, van de Donk N, Arnulf B (2022). Incidence and management of CAR-T neurotoxicity in patients with multiple myeloma treated with ciltacabtagene autoleucel in CARTITUDE studies. Blood Cancer J.

[191] Berdeja JG, Madduri D, Usmani SZ, Jakubowiak A, Agha M, Cohen AD (2021). Ciltacabtagene autoleucel, a B-cell maturation antigen-directed chimeric antigen receptor T-cell therapy in patients with relapsed or refractory multiple myeloma (CARTITUDE-1): a phase 1b/2 open-label study. Lancet.

[192] Abraham J (2020). Passive antibody therapy in COVID-19. Nat Rev Immunol.

[193] Kaplon H, Chenoweth A, Crescioli S, Reichert JM (2022). Antibodies to watch in 2022. mAbs..

[194] Orders M (2022). An EUA for bebtelovimab for treatment of COVID-19. Med Lett Drugs Ther.

[195] Efficacy and safety of two neutralising monoclonal antibody therapies, sotrovimab and BRII-196 plus BRII-198, for adults hospitalised with COVID-19 (TICO): a randomised controlled trial. Lancet Infect Dis. 2022;22(5):622–635. 10.1016/s1473-3099(21)00751-9 10.1016/S1473-3099(21)00751-9 PMC8700279 34953520

[196] Tixagevimab and cilgavimab (Evusheld) for pre-exposure prophylaxis of COVID-19. JAMA. 2022;327(4):384–385. 10.1001/jama.2021.24931 10.1001/jama.2021.24931 35076671

[197] Syed YY (2021). Regdanvimab: first approval. Drugs.

[198] Gupta A, Gonzalez-Rojas Y, Juarez E, Crespo Casal M, Moya J, Falci DR (2021). Early treatment for Covid-19 with SARS-CoV-2 neutralizing antibody sotrovimab. N Engl J Med.

[199] Deeks ED (2021). Casirivimab/imdevimab: first approval. Drugs.

[200] Lomakin NV, Bakirov BA, Protsenko DN, Mazurov VI, Musaev GH, Moiseeva OM (2021). The efficacy and safety of levilimab in severely ill COVID-19 patients not requiring mechanical ventilation: results of a multicenter randomized double-blind placebo-controlled phase III CORONA clinical study. Inflamm Res.

[201] Somers EC, Eschenauer GA, Troost JP, Golob JL, Gandhi TN, Wang L (2021). Tocilizumab for treatment of mechanically ventilated patients with COVID-19. Clin Infect Dis.

[202] Westendorf K, Žentelis S, Wang L, Foster D, Vaillancourt P, Wiggin M (2022). LY-CoV1404 (bebtelovimab) potently neutralizes SARS-CoV-2 variants. Cell Rep.

[203] Chen EC, Gilchuk P, Zost SJ, Suryadevara N, Winkler ES, Cabel CR (2021). Convergent antibody responses to the SARS-CoV-2 spike protein in convalescent and vaccinated individuals. Cell Rep.

[204] Center for Drug Evaluation, Research. FDA updates sotrovimab emergency use authorization. U.S. Food and Drug Administration. https://www.fda.gov/drugs/drug-safety-and-availability/fda-updates-sotrovimab-emergency-use-authorization. Accessed 31 May 2022

[205] Wang P, Nair MS, Liu L, Iketani S, Luo Y, Guo Y (2021). Antibody resistance of SARS-CoV-2 variants B.1.351 and B.1.1.7. Nature..

[206] Wang P, Casner RG, Nair MS, Wang M, Yu J, Cerutti G (2021). Increased resistance of SARS-CoV-2 variant P.1 to antibody neutralization. Cell Host Microbe..

[207] Chen RE, Winkler ES, Case JB, Aziati ID, Bricker TL, Joshi A (2021). In vivo monoclonal antibody efficacy against SARS-CoV-2 variant strains. Nature.

[208] Hoffmann M, Hofmann-Winkler H, Krüger N, Kempf A, Nehlmeier I, Graichen L (2021). SARS-CoV-2 variant B.1.617 is resistant to bamlanivimab and evades antibodies induced by infection and vaccination. Cell Rep..

[209] Focosi D, Novazzi F, Genoni A, Dentali F, Gasperina DD, Baj A (2021). Emergence of SARS-COV-2 spike protein escape mutation Q493R after treatment for COVID-19. Emerg Infect Dis.

[210] O’Brien MP, Forleo-Neto E, Musser BJ, Isa F, Chan K-C, Sarkar N (2021). Subcutaneous REGEN-COV antibody combination to prevent Covid-19. N Engl J Med.

[211] Weinreich DM, Sivapalasingam S, Norton T, Ali S, Gao H, Bhore R (2021). REGEN-COV antibody combination and outcomes in outpatients with Covid-19. N Engl J Med.

[212] Anderson TS, O’Donoghue A, Mechanic O, Dechen T, Stevens J (2022). Administration of anti–SARS-CoV-2 monoclonal antibodies after US Food and Drug Administration deauthorization. JAMA Netw Open.

[213] US Food and Drug Administration. Coronavirus (COVID-19) update: FDA limits use of certain monoclonal antibodies to treat COVID-19 due to the Omicron variant. January 24, 2022.. https://www.fda.gov/news-events/press-announcements/coronavirus-covid-19-update-fda-limits-use-certain-monoclonal-antibodies-treat-covid-19-due-omicron. Accessed 12 Mar 2022

[214] Chowdhury S, Bappy MH, Desai S, Chowdhury S, Patel V, Chowdhury MS, Fonseca A, Sekzer C, Zahid S, Patousis A, Gerothanasi A, Masenga MJ (2022). COVID-19 and pregnancy. Discoveries.

[215] Koshida S, Asanuma K, Kuribayashi K, Goto M, Tsuji N, Kobayashi D (2010). Prevalence of human anti-mouse antibodies (HAMAs) in routine examinations. Clin Chim Acta.

[216] Vande Casteele N, Gils A (2015). Pharmacokinetics of anti-TNF monoclonal antibodies in inflammatory bowel disease: adding value to current practice. J Clin Pharmacol.

[217] Baert F, Noman M, Vermeire S, Van Assche G, D'Haens G, Carbonez A (2003). Influence of immunogenicity on the long-term efficacy of infliximab in Crohn's disease. N Engl J Med..

[218] Dempsey PW, Allison MED, Akkaraju S, Goodnow CC, Fearon DT (1996). C3d of Complement as a molecular adjuvant: bridging innate and acquired immunity. Science.

[219] Chang JCC, Diveley JP, Savary JR, Jensen FC (1998). Adjuvant activity of incomplete Freund's adjuvant. Adv Drug Deliv Rev.

[220] McCluskie MJ, Weeratna RD, Krieg AM, Davis HL (2000). CpG DNA is an effective oral adjuvant to protein antigens in mice. Vaccine.

[221] Klinman DM, Barnhart KM, Conover J (1999). CpG motifs as immune adjuvants. Vaccine.

[222] Garg R, Babiuk L, van DrunenLittel-van den Hurk S, Gerdts V (2017). A novel combination adjuvant platform for human and animal vaccines. Vaccine..

[223] Hammond SA, Tsonis C, Sellins K, Rushlow K, Scharton-Kersten T, Colditz I (2000). Transcutaneous immunization of domestic animals: opportunities and challenges. Adv Drug Deliv Rev.

[224] Barbieri JT, Price BM, Liner AL, Park S, Leppla SH, Mateczun A (2001). Protection against anthrax lethal toxin challenge by genetic immunization with a plasmid encoding the lethal factor protein. Infect Immun.

[225] Jazayeri M, Soleimanjahi H, Fotouhi F, Pakravan N (2009). Comparison of intramuscular and footpad subcutaneous immunization with DNA vaccine encoding HSV-gD2 in mice. Comp Immunol Microbiol Infect Dis.

[226] Liu S, Wang S, Lu S (2016). DNA immunization as a technology platform for monoclonal antibody induction. Emerg Microbes Infect..

[227] Wang S, Zhang C, Zhang L, Li J, Huang Z, Lu S (2008). The relative immunogenicity of DNA vaccines delivered by the intramuscular needle injection, electroporation and gene gun methods. Vaccine.

[228] Tan P, Mitchell DA, Buss TN, Holmes MA, Anasetti C, Foote J (2002). “Superhumanized” antibodies: reduction of immunogenic potential by complementarity-determining region grafting with human germline sequences: application to an anti-CD28. J Immunol.

[229] Söderlind E, Strandberg L, Jirholt P, Kobayashi N, Alexeiva V, Åberg A-M (2000). Recombining germline-derived CDR sequences for creating diverse single-framework antibody libraries. Nat Biotechnol.

[230] Colaert N, Helsens K, Martens L, Vandekerckhove J, Gevaert K (2009). Improved visualization of protein consensus sequences by iceLogo. Nat Methods.

[231] Nault JC, Mallet M, Pilati C, Calderaro J, Bioulac-Sage P, Laurent C (2013). High frequency of telomerase reverse-transcriptase promoter somatic mutations in hepatocellular carcinoma and preneoplastic lesions. Nat Commun.

[232] Presta LG (2006). Engineering of therapeutic antibodies to minimize immunogenicity and optimize function. Adv Drug Deliv Rev.

[233] Antibody humanization methods for development of therapeutic applications. Monoclon Antib Immunodiagn Immunother. 2014;33(2):67-73. 10.1089/mab.2013.0080 10.1089/mab.2013.0080 24746146

[234] Kashmiri SVS, De Pascalis R, Gonzales NR, Schlom J (2005). SDR grafting—a new approach to antibody humanization. Methods.

[235] Olimpieri PP, Marcatili P, Tramontano A (2015). Tabhu: tools for antibody humanization. Bioinformatics.

[236] Prihoda D, Maamary J, Waight A, Juan V, Fayadat-Dilman L, Svozil D (2022). BioPhi: a platform for antibody design, humanization, and humanness evaluation based on natural antibody repertoires and deep learning. MAbs.

[237] Marks C, Hummer AM, Chin M, Deane CM (2021). Humanization of antibodies using a machine learning approach on large-scale repertoire data. Bioinformatics.

[238] Lefranc MP (2003). IMGT® databases, web resources and tools for immunoglobulin and T cell receptor sequence analysis, http://imgt.cines.fr. Leukemia..

[239] Smith GP (1985). Filamentous fusion phage: novel expression vectors that display cloned antigens on the virion surface. Science.

[240] Vaughan TJ, Williams AJ, Pritchard K, Osbourn JK, Pope AR, Earnshaw JC (1996). Human antibodies with sub-nanomolar affinities isolated from a large non-immunized phage display library. Nat Biotechnol.

[241] Peltomaa R, Benito-Peña E, Barderas R, Moreno-Bondi MC (2019). Phage display in the quest for new selective recognition elements for biosensors. ACS Omega..

[242] Nixon AE, Sexton DJ, Ladner RC (2014). Drugs derived from phage display: from candidate identification to clinical practice. MAbs.

[243] Lee CV, Liang W-C, Dennis MS, Eigenbrot C, Sidhu SS, Fuh G (2004). High-affinity human antibodies from phage-displayed synthetic Fab libraries with a single framework scaffold. J Mol Biol.

[244] Barbas CF, Kang AS, Lerner RA, Benkovic SJ (1991). Assembly of combinatorial antibody libraries on phage surfaces: the gene III site. Proc Natl Acad Sci U S A.

[245] Ahmad ZA, Yeap SK, Ali AM, Ho WY, Alitheen NBM, Hamid M (2012). ScFv antibody: principles and clinical application. Clin Dev Immunol.

[246] Sheets MD, Amersdorfer P, Finnern R, Sargent P, Lindqvist E, Schier R (1998). Efficient construction of a large nonimmune phage antibody library: the production of high-affinity human single-chain antibodies to protein antigens. Proc Natl Acad Sci U S A.

[247] Smolarek D, Bertrand O, Czerwinski M (2012). Variable fragments of heavy chain antibodies (VHHs): a new magic bullet molecule of medicine?. Postepy Hig Med Dosw (Online).

[248] Bradbury ARM, Sidhu S, Dübel S, McCafferty J (2011). Beyond natural antibodies: the power of in vitro display technologies. Nat Biotechnol.

[249] Lerner RA (2016). Combinatorial antibody libraries: new advances, new immunological insights. Nat Rev Immunol.

[250] Fellouse FA, Esaki K, Birtalan S, Raptis D, Cancasci VJ, Koide A (2007). High-throughput generation of synthetic antibodies from highly functional minimalist phage-displayed libraries. J Mol Biol.

[251] Chen G, Sidhu SS (2014). Design and generation of synthetic antibody libraries for phage display. Methods Mol Biol.

[252] Hammers CM, Stanley JR (2014). Antibody phage display: technique and applications. J Invest Dermatol.

[253] Haque A, Tonks NK (2012). The use of phage display to generate conformation-sensor recombinant antibodies. Nat Protoc.

[254] Saw PE, Song EW (2019). Phage display screening of therapeutic peptide for cancer targeting and therapy. Protein Cell.

[255] Ledsgaard L, Kilstrup M, Karatt-Vellatt A, McCafferty J, Laustsen AH (2018). Basics of antibody phage display technology. Toxins (Basel)..

[256] Lu R-M, Hwang Y-C, Liu IJ, Lee C-C, Tsai H-Z, Li H-J (2020). Development of therapeutic antibodies for the treatment of diseases. J Biomed Sci.

[257] Alt FW, Keith Blackwell T, Yancopoulos GD (1985). Immunoglobulin genes in transgenic mice. Trends Genet.

[258] Brüggemann M, Caskey HM, Teale C, Waldmann H, Williams GT, Surani MA (1989). A repertoire of monoclonal antibodies with human heavy chains from transgenic mice. Proc Natl Acad Sci U S A.

[259] Taylor LD, Carmack CE, Schramm SR, Mashayekh R, Higgins KM, Kuo CC (1992). A transgenic mouse that expresses a diversity of human sequence heavy and light chain immunoglobulins. Nucleic Acids Res.

[260] Chen J, Trounstine M, Alt FW, Young F, Kurahara C, Loring JF (1993). Immunoglobulin gene rearrangement in B cell deficient mice generated by targeted deletion of the JH locus. Int Immunol.

[261] Chen J, Trounstine M, Kurahara C, Young F, Kuo CC, Xu Y (1993). B cell development in mice that lack one or both immunoglobulin kappa light chain genes. EMBO J.

[262] Lonberg N, Taylor LD, Harding FA, Trounstine M, Higgins KM, Schramm SR (1994). Antigen-specific human antibodies from mice comprising four distinct genetic modifications. Nature.

[263] Jakobovits A, Amado RG, Yang X, Roskos L, Schwab G (2007). From XenoMouse technology to panitumumab, the first fully human antibody product from transgenic mice. Nat Biotechnol.

[264] Teng G, Papavasiliou FN (2007). Immunoglobulin somatic hypermutation. Annu Rev Genet.

[265] Hoffman W, Lakkis FG, Chalasani G (2016). B Cells, antibodies, and more. Clin J Am Soc Nephrol.

[266] Osborn MJ, Ma B, Avis S, Binnie A, Dilley J, Yang X (2013). High-affinity IgG antibodies develop naturally in Ig-knockout rats carrying germline human IgH/Igκ/Igλ loci bearing the rat CH Region. J Immunol.

[267] Hozumi N, Tonegawa S (1976). Evidence for somatic rearrangement of immunoglobulin genes coding for variable and constant regions. Proc Natl Acad Sci U S A.

[268] Brack C, Hirama M, Lenhard-Schuller R, Tonegawa S (1978). A complete immunoglobulin gene is created by somatic recombination. Cell.

[269] Rajpal A, Beyaz N, Haber L, Cappuccilli G, Yee H, Bhatt RR (2005). A general method for greatly improving the affinity of antibodies by using combinatorial libraries. Proc Natl Acad Sci U S A.

[270] Steidl S, Ratsch O, Brocks B, Dürr M, Thomassen-Wolf E (2008). In vitro affinity maturation of human GM-CSF antibodies by targeted CDR-diversification. Mol Immunol.

[271] Steinitz M, Klein G, Koskimies S, Makel O (1977). EB virus-induced B lymphocyte cell lines producing specific antibody. Nature.

[272] Tsujimoto Y (1989). Overexpression of the human BCL-2 gene product results in growth enhancement of Epstein-Barr virus-immortalized B cells. Proc Natl Acad Sci U S A.

[273] Tosato G, Tanner J, Jones KD, Revel M, Pike SE (1990). Identification of interleukin-6 as an autocrine growth factor for Epstein-Barr virus-immortalized B cells. J Virol.

[274] Effect of interleukins on antibody production by epstein-barr virus transformed B Cells. Monoclon Antib Immunodiagn Immunother. 2015;34(3):162–168. 10.1089/mab.2014.0067 10.1089/mab.2014.0067 26090593

[275] Smith K, Garman L, Wrammert J, Zheng N-Y, Capra JD, Ahmed R (2009). Rapid generation of fully human monoclonal antibodies specific to a vaccinating antigen. Nat Protoc.

[276] Jiang X, Suzuki H, Hanai Y, Wada F, Hitomi K, Yamane T (2006). A novel strategy for generation of monoclonal antibodies from single B cells using RT-PCR technique and in vitro expression. Biotechnol Prog.

[277] Tiller T, Meffre E, Yurasov S, Tsuiji M, Nussenzweig MC, Wardemann H (2008). Efficient generation of monoclonal antibodies from single human B cells by single cell RT-PCR and expression vector cloning. J Immunol Methods.

[278] Shi Z, Zhang Q, Yan H, Yang Y, Wang P, Zhang Y (2019). More than one antibody of individual B cells revealed by single-cell immune profiling. Cell Discov..

[279] Macosko Evan Z, Basu A, Satija R, Nemesh J, Shekhar K, Goldman M (2015). Highly parallel genome-wide expression profiling of individual cells using nanoliter droplets. Cell.

[280] Klein Allon M, Mazutis L, Akartuna I, Tallapragada N, Veres A, Li V (2015). Droplet barcoding for single-cell transcriptomics applied to embryonic stem cells. Cell.

[281] Wardemann H, Yurasov S, Schaefer A, Young JW, Meffre E, Nussenzweig MC (2003). Predominant autoantibody production by early human B cell precursors. Science.

[282] Ju B, Zhang Q, Ge J, Wang R, Sun J, Ge X (2020). Human neutralizing antibodies elicited by SARS-CoV-2 infection. Nature.

[283] An EUA (2021). for bamlanivimab and etesevimab for COVID-19. Med Lett Drugs Ther.

[284] Gottlieb RL, Nirula A, Chen P, Boscia J, Heller B, Morris J (2021). Effect of bamlanivimab as monotherapy or in combination with etesevimab on viral load in patients with mild to moderate COVID-19: a randomized clinical trial. JAMA.

[285] Dougan M, Nirula A, Azizad M, Mocherla B, Gottlieb RL, Chen P (2021). Bamlanivimab plus etesevimab in mild or moderate Covid-19. N Engl J Med.

[286] Nathan R, Shawa I, De La Torre I, Pustizzi JM, Haustrup N, Patel DR (2021). A narrative review of the clinical practicalities of bamlanivimab and etesevimab antibody therapies for SARS-CoV-2. Infect Dis Ther.

[287] Heo Y-A (2022). Sotrovimab: first approval. Drugs.

[288] Gupta A, Gonzalez-Rojas Y, Juarez E, Crespo Casal M, Moya J, Falci DR (2021). Early treatment for Covid-19 with SARS-CoV-2 neutralizing antibody sotrovimab. N Engl J Med.

[289] Jumper J, Evans R, Pritzel A, Green T, Figurnov M, Ronneberger O (2021). Highly accurate protein structure prediction with AlphaFold. Nature.

[290] Varadi M, Anyango S, Deshpande M, Nair S, Natassia C, Yordanova G (2022). AlphaFold protein structure database: massively expanding the structural coverage of protein-sequence space with high-accuracy models. Nucleic Acids Res.

[291] Baek M, DiMaio F, Anishchenko I, Dauparas J, Ovchinnikov S, Lee GR (2021). Accurate prediction of protein structures and interactions using a three-track neural network. Science.

[292] Pennisi E (2021). Protein structure prediction now easier, faster. Science.

[293] Baek M, Baker D (2022). Deep learning and protein structure modeling. Nat Methods.

[294] Cramer P (2021). AlphaFold2 and the future of structural biology. Nat Struct Mol Biol.

[295] Ruffolo JA, Sulam J, Gray JJ (2022). Antibody structure prediction using interpretable deep learning. Patterns.

[296] Schneider C, Buchanan A, Taddese B, Deane CM (2021). DLAB-Deep learning methods for structure-based virtual screening of antibodies. Bioinformatics.

[297] Abanades B, Georges G, Bujotzek A, Deane CM (2022). ABlooper: Fast accurate antibody CDR loop structure prediction with accuracy estimation. Bioinformatics.

[298] Akbar R, Robert PA, Pavlović M, Jeliazkov JR, Snapkov I, Slabodkin A (2021). A compact vocabulary of paratope-epitope interactions enables predictability of antibody-antigen binding. Cell Rep.

[299] Mason DM, Friedensohn S, Weber CR, Jordi C, Wagner B, Meng SM (2021). Optimization of therapeutic antibodies by predicting antigen specificity from antibody sequence via deep learning. Nat Biomed Eng.

[300] Shan S, Luo S, Yang Z, Hong J, Su Y, Ding F (2022). Deep learning guided optimization of human antibody against SARS-CoV-2 variants with broad neutralization. Proc Natl Acad Sci U S A.

[301] Cao L, Coventry B, Goreshnik I, Huang B, Sheffler W, Park JS (2022). Design of protein-binding proteins from the target structure alone. Nature.

[302] Liu Z, Gunasekaran K, Wang W, Razinkov V, Sekirov L, Leng E (2014). Asymmetrical Fc engineering greatly enhances antibody-dependent cellular cytotoxicity (ADCC) effector function and stability of the modified antibodies. J Biol Chem.

[303] Mimura Y, Katoh T, Saldova R, O’Flaherty R, Izumi T, Mimura-Kimura Y (2018). Glycosylation engineering of therapeutic IgG antibodies: challenges for the safety, functionality and efficacy. Protein Cell.

[304] Li T, DiLillo DJ, Bournazos S, Giddens JP, Ravetch JV, Wang L-X (2017). Modulating IgG effector function by Fc glycan engineering. Proc Natl Acad Sci U S A.

[305] Chen C-L, Hsu J-C, Lin C-W, Wang C-H, Tsai M-H, Wu C-Y (2017). Crystal structure of a homogeneous IgG-Fc glycoform with the N-glycan designed to maximize the antibody dependent cellular cytotoxicity. ACS Chem Biol.

[306] Saunders KO (2019). Conceptual approaches to modulating antibody effector functions and circulation half-life. Front Immunol.

[307] Papadopoulos N, Martin J, Ruan Q, Rafique A, Rosconi MP, Shi E (2012). Binding and neutralization of vascular endothelial growth factor (VEGF) and related ligands by VEGF Trap, ranibizumab and bevacizumab. Angiogenesis.

[308] Sharabi AB, Lim M, DeWeese TL, Drake CG (2015). Radiation and checkpoint blockade immunotherapy: radiosensitisation and potential mechanisms of synergy. Lancet Oncol.

[309] de Goeij BECG, Vink T, ten Napel H, Breij ECW, Satijn D, Wubbolts R (2016). Efficient payload delivery by a bispecific antibody-drug conjugate targeting HER2 and CD63. Mol Cancer Ther.

[310] Li X, Song Y (2020). Proteolysis-targeting chimera (PROTAC) for targeted protein degradation and cancer therapy. J Hematol Oncol.

[311] Békés M, Langley DR, Crews CM (2022). PROTAC targeted protein degraders: the past is prologue. Nat Rev Drug Discov.

[312] Dragovich PS (2022). Degrader-antibody conjugates. Chem Soc Rev.

[313] Abhinandan KR, Martin ACR (2007). Analyzing the “degree of humanness” of antibody sequences. J Mol Biol.

