# Supplementary material for: Development of therapeutic antibodies for the treatment of diseases
Source: Mol Biomed. 2022 Nov 22;3:35. doi: 10.1186/s43556-022-00100-4 (PMC9684400; doi:10.1186/s43556-022-00100-4)
Supplement: Supplementary file 1 — Additional file 1: Supplementary Table 1. Monoclonal antibodies in late-stage clinical trialsa. [file 43556_2022_100_MOESM1_ESM.docx]

Supplementary Table 1. Monoclonal antibodies in late-stage clinical trials^a^.

| Therapeutic Area | INN^*^ | Drug Code(s)^*^ | Target | Phase of Clinical Development | Late-stage clinical study^b^ |
| --- | --- | --- | --- | --- | --- |
| Oncology | Zolbetuximab | iMAB362 | Claudin18.2 | Phase Ⅲ | (NCT03504397; NCT03653507) |
| Oncology | Zilovertamab | UC-961 | ROR1 | Phase Ⅲ pending | (NCT05431179) |
| Oncology | Zalifrelimab | AGEN1884 | CTLA-4 | Phase Ⅱ (pivotal) | (NCT03894215) |
| Oncology | Vibostolimab | MK-7684 | TIGIT | Phase Ⅲ | (NCT04738487) |
| Oncology | Tiragolumab | MTIG7192A, RO7092284, RG6058 | TIGIT | Phase Ⅲ | (NCT04294810; NCT04513925; (NCT04543617; NCT04540211;  (NCT04665856; NCT04256421) |
| Oncology | Sasanlimab | PF-06801591 | PD-1 | Phase Ⅲ | (NCT04165317) |
| Oncology | Sabatolimab | MBG453 | TIM-3 | Phase Ⅲ | (NCT04266301) |
| Oncology | Rulonilimab | F520 | PD-1 | Phase Ⅲ pending | (NCT05408221) |
| Oncology | Quavonlimab | MK-1308, AK107 | CTLA-4 | Phase Ⅲ | (NCT04736706) |
| Oncology | Oregovomab | Mab-B43.13 | CA125 | Phase Ⅲ | (NCT04498117) |
| Oncology | Oleclumab | MEDI9447 | CD73 | Phase Ⅲ | (NCT05221840) |
| Oncology | Ociperlimab | BGB-A1217 | TIGIT | Phase Ⅲ | (NCT04746924; NCT04866017) |
| Oncology | Nofazinlimab | CS1003 | PD-1 | Phase Ⅲ | (NCT04194775) |
| Oncology | Nadunolimab | CAN04 | IL1R Accessory Protein | Phase Ⅱ/Ⅲ  pending | (NCT04229004) |
| Oncology | Monalizumab | NN8765, NNC141-0100 | NKG2A | Phase Ⅲ | (NCT04590963) |
| Oncology | Ivuxolimab | PF-04518600 | OX40 | Phase Ⅲ | (NCT05059522) |
| Oncology | Finotonlimab | SCTI-10A | PD-1 | Phase Ⅱ/Ⅲ and  Phase Ⅲ | (NCT04560894;  NCT04171284;  NCT04146402) |
| Oncology | Fianlimab | REGN3767 | LAG-3 | Phase Ⅲ | (NCT05352672) |
| Oncology | Felzartamab | TJ202, MOR202, MOR03087 | CD38 | Phase Ⅲ | (NCT03952091) |
| Oncology | Favezelimab | MK-4280 | LAG-3 | Phase Ⅲ | (NCT05064059) |
| Oncology | Emactuzumab | RO5509554, RG7155 | CD115 | Phase Ⅲ pending | (NCT05417789) |
| Oncology | Domvanalimab | AB154 | TIGIT | Phase Ⅲ | (NCT04736173) |
| Oncology | Cosibelimab | CK-301 | PD-L1 | Phase Ⅲ | (NCT03212404)  (NCT04786964) |
| Oncology | Coprelotamab | GB221 | HER-2 | Phase Ⅲ | (NCT04164615) |
| Oncology | Cobolimab | GSK4069889, TSR-022 | TIM-3 | Phase Ⅱ/Ⅲ | (NCT04655976) |
| Oncology | Cetrelimab | JNJ-63723283 | PD-1 | Phase Ⅱ/Ⅲ and Phase Ⅲ | (NCT03357952)  (NCT04658862) |
| Oncology | Bemarituzumab | FPA144 | FGFR2b | Phase Ⅲ | (NCT05052801) |
| Oncology | Adebrelimab | SHR-1316, HTI-1088 | PD-L1 | Phase Ⅲ | (NCT04316364)  (NCT04691063) |
| Oncology | - | APX003, BD0801, TK001, | VEGF | Phase Ⅲ | (NCT04908787) |
| Oncology | - | CBT-502, TQB2450, APL-502 | PD-L1 | Phase Ⅲ | (NCT04523272) NCT04325763, NCT04964479) (NCT03855384)  (NCT04809142) |
| Oncology | - | IBI310 | CTLA-4 | Phase Ⅲ | (NCT04277663)  (NCT04720716) |
| Oncology | - | JY-025 | VEGFR2 | Phase Ⅱ/Ⅲ  pending | (NCT04874844) |
| Oncology | - | MIL62 | CD20 | Phase Ⅲ | (NCT04834024) |
| Oncology | - | NIS793, XOMA-089 | TGF beta 1 and 2 | Phase Ⅲ | (NCT04935359) |
| Oncology | - | QL1604 | PD-1 | Phase Ⅱ/Ⅲ | (NCT04864782) |
| Immunology | Xeligekimab | GR1501 | IL-17A | Phase Ⅲ | (ChiCTR2100043223) |
| Immunology | Vunakizumab | SHR-1314 | IL-17A | Phase Ⅱ/Ⅲ | (NCT04840485) (NCT04839016) |
| Immunology | Suciraslimab | SM03 | CD22 | Phase Ⅲ | (NCT04312815) |
| Immunology | Sibeprenlimab | VIS649 | APRIL | Phase Ⅲ | (NCT05248646; NCT05248659) |
| Immunology | Rozanolixizumab | UCB7665 | FcRn | Phase Ⅲ | (NCT03971422, NCT04124965, NCT04650854)  (NCT04200456, NCT04224688, NCT04596995) |
| Immunology | Rocatinlimab | KHK4083, AMG451 | OX40 | Phase Ⅲ | (NCT05398445) |
| Immunology | Otilimab | GSK3196165, MOR103, MOR-04357 | GM-CSF | Phase Ⅲ | (NCT03980483, NCT03970837, NCT04134728, NCT04333147) |
| Immunology | Nipocalimab | M281 | FcRn | Phase Ⅱ/Ⅲ and Phase Ⅲ | (NCT04119050)  (NCT04951622) |
| Immunology | Litifilimab | BIIB059 | Blood dendritic  cell antigen 2 | Phase Ⅲ | (NCT04961567, NCT04895241) |
| Immunology | Ligelizumab | QGE031 | IgE | Phase Ⅲ | (NCT03580356, NCT03580369, NCT03907878, NCT04210843) (NCT05024058) (NCT04984876) |
| Immunology | Lebrikizumab | RG3637,  TNX-650, PRO301444, MILR1444A | IL-13 | Phase Ⅲ | (NCT04250337, NCT04760314, NCT04146363, NCT04178967, NCT04626297, NCT04392154, NCT04250350, NCT05559359) |
| Immunology | Imsidolimab | ANB019 | IL-36R | Phase Ⅲ | (GEMINI-1) |
| Immunology | Ianalumab | VAY736 | BLyS/BAFF/TACI/BCMA receptor | Phase Ⅱ/Ⅲ and Phase Ⅲ | (NCT03217422)  (NCT05126277) |
| Immunology | Ebdarokimab | AK101 | IL-12/23p40 | Phase Ⅲ | (NCT05120297) |
| Immunology | Divozilimab | BCD-132 | CD20 | Phase Ⅲ | (NCT05385744) |
| Immunology | Depemokimab | GSK3511294 | IL-5 | Phase Ⅲ | (NCT04719832, NCT04718103, NCT04718389) |
| Immunology | Clazakizumab | ALD518,  BMS-945429 | IL-6 | Phase Ⅲ | (NCT03744910) |
| Immunology | Cendakimab | CC-93538, RPC4046; ABT-308 | IL-13 | Phase Ⅲ | (NCT04753697, NCT04991935) |
| Immunology | Brazikumab | AMG139, MEDI2070 | IL-23 | Phase Ⅱ/Ⅲ and Phase Ⅲ | (NCT03759288, NCT03961815) |
| Immunology | Birtamimab | NEOD001 | Amyloid | Phase Ⅲ | (NCT04973137) |
| Immunology | Batoclimab | IMVT-1401, RVT-1401, HL161BKN, HBM9161, HL161 | FcRn | Phase Ⅲ | (NCT04428255)  (NCT05015127)  (NCT05039190) |
| Immunology | Anselamimab | CAEL-101, ChmAb11-1F4 | Amyloid fibril | Phase Ⅲ | (NCT04512235, NCT04504825) |
| Infectious disease | Upanovimab | SCTA01 | SARS-CoV-2 | Phase Ⅱ/Ⅲ | (NCT04644185) |
| Infectious disease | Tosatoxumab | AR-301, KBSA301 | S. aureus alpha-toxin | Phase Ⅲ | (NCT03816956) |
| Infectious disease | Suvratoxumab | AR-320, MEDI4893 | Staphylococcus aureus alpha toxin | Phase Ⅲ pending | (NCT05331885 pending) |
| Infectious disease | Semzuvolimab | UB-421,  dB4C7 | CD4 | Phase Ⅲ | (NCT04406727, NCT03149211) |
| Infectious disease | Plonmarlimab | TJ003234, TJM2 | GM-CSF | Phase Ⅱ/Ⅲ | (NCT04341116) |
| Infectious disease | Clesrovimab | MK-1654 | RSV fusion  protein | Phase Ⅱ/Ⅲ and Phase Ⅲ | (NCT04767373, NCT04938830) |
| Infectious disease | - | MAD0004J08 | SARS-CoV-2 | Phase Ⅱ/Ⅲ | (NCT04952805) |
| Cardiovascular/hemostasis | Ziltivekimab | COR-001 | IL-6 | Phase Ⅲ | (NCT05021835) |
| Cardiovascular/hemostasis | Recaticimab | SHR-1209 | PCSK9 | Phase Ⅲ | (NCT04849000, NCT04885218)  (NCT04844125) |
| Cardiovascular/hemostasis | Pozelimab | REGN3918 | Complement C5 | Phase Ⅱ/Ⅲ and Ⅲ | (NCT04209634)  (NCT04162470)  (NCT05070858) |
| Cardiovascular/hemostasis | Ongericimab | JS002 | PCSK9 | Phase Ⅲ | (NCT04781114) |
| Cardiovascular/hemostasis | Marstacimab | PF-06741086 | Tissue factor  pathway inhibitor | Phase Ⅲ | (NCT03938792) |
| Cardiovascular/hemostasis | Inclacumab | RO4905417, RG1512 | P-selectin (CD62) | Phase Ⅲ | (NCT04927247, NCT04935879) |
| Cardiovascular/hemostasis | Garadacimab | CSL312 | Factor XIIa | Phase Ⅲ | (NCT04656418, NCT04739059) |
| Cardiovascular/hemostasis | Ebronucimab | AK102 | PCSK9 | Phase Ⅲ | (NCT05255094, NCT05260411) |
| Cardiovascular/hemostasis | Concizumab | NNC172-2021, NN7415 | Tissue factor pathway inhibitor | Phase Ⅲ | (NCT04082429) |
| Cardiovascular/hemostasis | Abelacimab | MAA868 | Factor XI | Phase Ⅲ | (NCT05171075, NCT05171049) |
| Cardiovascular/hemostasis | - | ANX005 | Complement C1q | Phase Ⅱ/Ⅲ | (NCT04701164) |
| Neurological disorders | Solanezumab | LY-2062430, hM266.2 | Amyloid beta, soluble | Phase Ⅱ/Ⅲ and Phase Ⅲ | (NCT01760005) (NCT02008357) |
| Neurological disorders | Latozinemab | AL001 | Sortilin | Phase Ⅲ | (NCT04374136) |
| Neurological disorders | Gantenerumab | RO4909832, RG1450 | Amyloid beta | Phase Ⅲ | (NCT01760005, NCT03444870, NCT03443973) (NCT04339413, NCT04374253) |
| Neurological disorders | Fasinumab | REGN475, SAR164877, MT-5547 | Nerve growth  factor | Phase Ⅲ | (NCT03161093, NCT02683239, NCT03304379, NCT02447276, NCT03245008)  (NCT02620020) |
| Neurological disorders | - | E2814 | Tau | Phase Ⅱ/Ⅲ | (NCT05269394) |
| Musculoskeletal disorders | Setrusumab | UX143, BPS804, MOR05813 | Sclerostin | Phase Ⅱ/Ⅲ | (NCT05125809) |
| Musculoskeletal disorders | Pamrevlumab | FG-3019 | Connective tissue growth factor | Phase Ⅲ | (NCT04632940, NCT04371666)  (NCT04419558, NCT03955146)  (NCT03941093, NCT04229004) |
| Musculoskeletal disorders | Garetosmab | REGN2477 | Activin A | Phase Ⅱ (pivotal); Phase Ⅲ pending | (NCT03188666; NCT05394116) |
| Musculoskeletal disorders | Apitegromab | SRK-015 | Myostatin | Phase Ⅲ | (NCT05156320) |
| Musculoskeletal disorders | - | RO7204239, GYM329, RG6237 | Myostatin | Phase Ⅱ/Ⅲ | (NCT05115110) |
| Respiratory | Tozorakimab | MEDI3506 | IL-33 | Phase Ⅲ | (NCT05166889, NCT05158387 not yet recruiting) |
| Respiratory | Paridiprubart | EB05,  NI-0101 | Toll-like receptor 4 | Phase Ⅱ/Ⅲ | (NCT04401475) |
| Respiratory | Lenzilumab | - | GM-CSF | Phase Ⅲ | (NCT04351152, NCT04583969) |
| Respiratory | Itepekimab | REGN3500, SAR440340 | IL-33 | Phase Ⅲ | (NCT04751487, NCT04701983) |
| Hematological Disorders | Vilobelimab | BDB-001,  IFX-1, CaCP29 | C5 | Phase Ⅲ | (NCT04449588) |

^*^Some antibodies have not been found INN (International Non-Proprietary Name) or Drug Code.

^a^Table data based on publicly available The Antibody Society ([www.antibodysociety.org/antibody-therapeutics-product-data/](http://www.antibodysociety.org/antibody-therapeutics-product-data/).) and ClinicalTrials.gov ([https://clinicaltrials.gov/).](https://clinicaltrials.gov/).%20%20%20%20b) ^b^NCT number: ClinicalTrials.gov identifier.
