# Supplementary material for: Development of therapeutic antibodies for the treatment of diseases
Source: Mol Biomed. 2022 Nov 22;3:35. doi: 10.1186/s43556-022-00100-4 (PMC9684400; doi:10.1186/s43556-022-00100-4)
Supplement: Supplementary file 2 — Additional file 2: Supplementary Table 2. Antibody fragments first approved or in late-stage clinical trialsa. [file 43556_2022_100_MOESM2_ESM.docx]

Supplementary Table 2. Antibody fragments first approved or in late-stage clinical trials^a^

| INN^*^ | Drug Code(s)^*^ | Target | Format | First global approval or  in late-stage clinical trials^a^ |
| --- | --- | --- | --- | --- |
| Abciximab | Reopro | GPIIb/IIIa | Fab | US, 1994 |
| Ranibizumab | Lucentis | VEG | Fab | US, 2006 |
| Certolizumab pegol | Cimzia | TNF | PEGylated Fab | US, 2008 |
| Idarucizumab | Praxbin | Dabigatran | Fab | US, 2015 |
| Moxetumomab pasudotox | Lumoxit | CD22 | dsFv immunotoxin | US, 2018 |
| Caplacizumab | Cablivi | von Willebrand factor | Nanobody | EU, 2018 |
| Brolucizumab | Beovu | VEGF-A | scFv | US, 2019 |
| Oportuzumab monatox | (Pending) | EpCAM | scFv immunotoxin | In review |
| Tebentafus | KIMMTRAK | gp100, CD3 | TCR-scFv fusion protein | US, 2022 |
| Envafolimab | ENWEIDA | PD-L1 | Single domain antibody-Fc | China, 2021 |
| Ozoralizumab | - | TNF, albumin | Nanobody | Regulatory review in Japan |
| Tebotelimab | MGD013 | PD, LAG-3 | Tandem domain-exchanged Fv | Phase Ⅱ/Ⅲ (NCT04082364) |
| Izalontamab | SI-B001 | EGFR, HER3 | IgG-(scFv)2 | Phase Ⅱ/Ⅲ (NCT05020457) |
| Glenzocimab | ACT017 | Glycoprotein VI on platelets | Humanized Fab | Phase Ⅱ/Ⅲ (NCT05070260) |
| Dapirolizumab pegol | CDP-7657 | CD40L | Humanized Fab | Phase Ⅲ (NCT04976322; NCT04294667) |
| Bentracimab | PB2452 | Ticagrelor | Human Fab | Phase Ⅲ (NCT04286438) |
| Glenzocimab | ACT-017 | GP6 | Fab | Phase Ⅱ/Ⅲ (NCT05559398) |
| Ranibizumab | - | VEGF | Fab | Phase Ⅲ (NCT05126966) |
| - | INBRX-109 | DR5 | sdAb (heavy chain only fused to Fc | Phase Ⅱ (potentially pivotal) (NCT04950075) |
| Sonelokimab | ALX 0761, | IL-17 | Nanobody | Phase Ⅱ (NCT03384745) |
| - | GSK 2862277 | TNFRSF1A | Nanobody | Phase Ⅱ (NCT02221037; NCT01818024) |
| - | BI-836880 | VEGF, ANGPT2 | Nanobody | Phase Ⅱ (NCT03861234) |
| Vobarilizumab | ALX 0061 | IL6RA | Nanobody | Phase Ⅱ (NCT02518620) |

*Some antibodies have not been found INN (International Non-Proprietary Name) or Drug Code.

^a^Table data based on publicly available The Antibody Society ([www.antibodysociety.org/antibody-therapeutics-product-data/](http://www.antibodysociety.org/antibody-therapeutics-product-data/).) and ClinicalTrials.gov ([https://clinicaltrials.gov/).](https://clinicaltrials.gov/).%20%20%20%20b) ^b^NCT number: ClinicalTrials.gov identifier.
